# Temporally and functionally distinct large-scale brain network dynamics supporting task switching

**DOI:** 10.1016/j.neuroimage.2022.119126

**Published:** 2022-03-22

**Authors:** Takumi Mitsuhashi, Masaki Sonoda, Ethan Firestone, Kazuki Sakakura, Jeong-Won Jeong, Aimee F. Luat, Sandeep Sood, Eishi Asano

**Affiliations:** a Department of Pediatrics, Children’s Hospital of Michigan, Detroit Medical Center, Wayne State University, 3901 Beaubien St, Detroit, MI 48201, USA; b Department of Neurology, Children’s Hospital of Michigan, Detroit Medical Center, Wayne State University, Detroit, MI 48201, USA; c Department of Neurosurgery, Children’s Hospital of Michigan, Detroit Medical Center, Wayne State University, Detroit, MI 48201, USA; d Department of Physiology, Wayne State University, Detroit, MI 48201, USA; e Department of Neurosurgery, Juntendo University, Tokyo 113-8421, Japan; f Department of Neurosurgery, Yokohama City University, Yokohama 236-0004, Japan; g Department of Neurosurgery, University of Tsukuba, Tsukuba 305-8575, Japan; h Department of Pediatrics, Central Michigan University, Mount Pleasant, MI 48858, USA

**Keywords:** Synchronization, Epilepsy surgery, Responses, Electrocorticography, *Ebb and Flow*, Cognitive control

## Abstract

**Objective:**

Our daily activities require frequent switches among competing responses at the millisecond time scale. We determined the spatiotemporal characteristics and functional significance of rapid, large-scale brain network dynamics during task switching.

**Methods:**

This cross-sectional study investigated patients with drug-resistant focal epilepsy who played a Lumosity cognitive flexibility training game during intracranial electroencephalography (iEEG) recording. According to a given task rule, unpredictably switching across trials, participants had to swipe the screen in the direction the stimulus was pointing or moving. Using this data, we described the spatiotemporal characteristics of iEEG high-gamma augmentation occurring more intensely during switch than repeat trials, unattributable to the effect of task rule (pointing or moving), within-stimulus congruence (the direction of stimulus pointing and moving was same or different in a given trial), or accuracy of an immediately preceding response. Diffusion-weighted imaging (DWI) tractography determined whether distant cortical regions showing enhanced activation during task switch trials were directly connected by white matter tracts. Trial-by-trial iEEG analysis deduced whether the intensity of task switch-related high-gamma augmentation was altered through practice and whether high-gamma amplitude predicted the accuracy of an upcoming response among switch trials.

**Results:**

The average number of completed trials during five-minute gameplay was 221.4 per patient (range: 171–285). Task switch trials increased the response times, whereas later trials reduced them. Analysis of iEEG signals sampled from 860 brain sites effectively elucidated the distinct spatiotemporal characteristics of task switch, task rule, and post-error-specific high-gamma modulations. Post-cue, task switch-related high-gamma augmentation was initiated in the right calcarine cortex after 260 ms, right precuneus after 330 ms, right entorhinal after 420 ms, and bilateral anterior middle-frontal gyri after 450 ms. DWI tractography successfully showed the presence of direct white matter tracts connecting the right visual areas to the precuneus and anterior middle-frontal regions but not between the right precuneus and anterior middle-frontal regions. Task-related high-gamma amplitudes in later trials were reduced in the calcarine, entorhinal and anterior middle-frontal regions, but increased in the precuneus. Functionally, enhanced post-cue precuneus high-gamma augmentation improved the accuracy of subsequent responses among switch trials.

**Conclusions:**

Our multimodal analysis uncovered two temporally and functionally distinct network dynamics supporting task switching. High-gamma augmentation in the visual-precuneus pathway may reflect the neural process facilitating an attentional shift to a given updated task rule. High-gamma activity in the visual-dorsolateral prefrontal pathway, rapidly reduced through practice, may reflect the cost of executing appropriate stimulus-response translation.

## 1. Introduction

At the millisecond scale, our daily activities require frequent switches among competing responses (Monsell, 2003), such as braking and handling while driving in a traffic jam. When task switching is required, it is challenging to execute correct motor output, but this response is improved with practice (Monsell, 2003; Fröber and Dreisbach, 2017; Steyvers et al., 2019; Steyvers and Schafer, 2020). The present study aimed to clarify the rapid, large-scale brain network dynamics involved in task switching. Collective evidence from lesioning, imaging, and electrophysiology studies suggests that the prefrontal and parietal cortices play causal roles in task switching. For example, frontal or parietal lobe lesions, regardless of hemisphere, were reported to impair task switching (Rogers et al., 1998; Stuss et al., 2001; Rushworth et al., 2003; Aron et al., 2004; Gläscher et al., 2012; Kopp et al., 2015; Arbula et al., 2021). Furthermore, studies of healthy adults using functional magnetic resonance imaging (fMRI) localized the cortical regions involved in task switching. Task switching trials, compared to repetition, were associated with more intense hemodynamic activations in large-scale brain regions, including the dorsolateral prefrontal areas (Dove et al., 2000; Sohn et al., 2000; Braver et al., 2003; Badre and Wagner, 2006; Yeung et al., 2006; Karayanidis et al., 2010; Kim et al., 2012; Jamadar et al., 2015; Vallesi et al., 2015; Cole et al., 2016) and medial parietal regions, on either hemisphere (Dove et al., 2000; Crone et al., 2006; Kim et al., 2012; von der Gablentz et al., 2015; Worringer et al., 2019). Invasive studies of non-human primates reported that prefrontal and parietal neurons were commonly activated during response preparation in task switch trials, but the spatial sampling was limited in these studies, making it challenging to identify network-wide modulations (Johnston et al., 2007; Phillips et al., 2014). Circumventing this issue, studies of healthy adults using scalp electroencephalography (EEG) reported that event-related potential (ERP) amplitudes at 300-ms post-cue differed between switch and repetition trials (Swainson et al., 2003; Kieffaber and Hetrick, 2005; Gajewski et al., 2010, 2018). However, such noninvasive electrophysiology studies were not designed to clarify the spatiotemporal order of local neural modulations (i.e., activation or suppression) occurring across the large-scale cortical networks.

Our present study visualized the neural dynamics involved in task switching using intracranial electroencephalography (iEEG). Event-related high-gamma activity at 70–110 Hz determined the spatiotemporal order of neural modulations while a given participant played a cognitive training game requiring occasional task switching. High-gamma activity is an outstanding biomarker of neural activity, capable of tracking millisecond-scale fluctuations (Shmuel et al., 2006; Crone et al., 2011; Lachaux et al., 2012; Miller et al., 2014). Augmentation of high gamma activity is associated with increased neural firing (Ray et al., 2008; Manning et al., 2009), hemodynamic responses (Scheeringa et al., 2011; Hermes et al., 2012), and glucose metabolism (Nishida et al., 2008). Notably, the iEEG’s outstanding signal fidelity (Ball et al., 2009) makes trial-by-trial high-gamma analysis possible at an individual-patient level (Vidal et al., 2012; Dastjerdi et al., 2013; Nourski et al., 2013; Uematsu et al., 2013; Coon and Schalk, 2016; Forseth et al., 2020). Considering the various iEEG frequency bands ranging from alpha through high-gamma, event-related high-gamma augmentation was reported to *best* predict the severity of cognitive deficits following focal brain resection (Sonoda et al., 2022). Diffusion-weighted imaging (DWI) tractography can visualize the direct white matter tracts allowing neural communications between distant cortical regions concurrently involved in a cognitive process (Sonoda et al., 2021). The present study elucidated the spatiotemporal dynamics and functional significance of iEEG-derived neural modulations during task switching by addressing the following aims.

[Aim 1: Temporal order of switch-related neural activations] We initially clarified the relative timing of local neural activations specifically involved in task switching at the millisecond time scale. Unlike scalp recording, iEEG allowed us to measure neural modulations directly from deep cortices such as the precuneus, a part of the medial parietal lobe region. This analysis successfully tested the hypothesis that high-gamma augmentation specific to switch trials would initially arise in the parietal cortex, followed by prefrontal areas. This hypothesis was partly motivated by the electrophysiological studies of non-human primates mentioned above (Johnston et al., 2007; Phillips et al., 2014). We also considered functional imaging studies of healthy human adults suggesting that the parietal cortices support the control or shift of spatial attention based on the visually cued context (Behrmann et al., 2004; Sato et al., 2010; Whitlock, 2017), whereas the prefrontal regions support the subsequent stimulus-response translation, including context-based decision (Brass et al., 2003; Koechlin et al., 2003; Sohn et al., 2007; Nee and D’Esposito, 2017).

[Aim 2: Association between behavioral priming and switch-related neural activation] We aimed to localize functionally distinct, task switch-related brain regions, in each of which event-related high-gamma activity was expected to be differentially correlated to practice-related behavioral improvement. Our principal hypothesis was that the neural cost, as quantified by high-gamma amplitude, would be extensively reduced in later trials, due to practice effects. This hypothesis was inspired by the recurrent behavioral observations that learning through practice allows humans to make relevant, rapid responses in a progressively effortless manner (Monsell, 2003; Steyvers et al., 2019; Steyvers and Schafer, 2020). Previous iEEG studies reported that repeated visual stimuli were correlated with a progressive reduction of high-gamma augmentation at perceptual (Matsuzaki et al., 2012; Eliades et al., 2014; Engell and McCarthy, 2014; Merzagora et al., 2014; Vidal et al., 2014; Rangarajan et al., 2020) and prefrontal areas (Korzeniewska et al., 2020). Such phenomenon of reduced neural responses to repeated stimuli is referred to as *repetition suppression* or neural adaptation (Summerfield et al., 2008; Khalighinejad et al., 2019). Conversely, we expected our iEEG analysis to localize small, distinct regions showing increased neural responses in later trials (i.e., *repetition enhancement*). To that point, a meta-analysis of 137 fMRI studies (Kim, 2017) reports that repetition enhancement is common in the precuneus region, which is suggested to support the allocation of spatial attention based on a task rule update (Shulman et al., 2009; Tosoni et al., 2013; Vandenberghe and Gillebert, 2009).

[Aim 3: Direct connectivity between distant cortices showing switch-related neural activation] We determined whether DWI-based white matter tracts existed between cortical regions showing switch-related high-gamma augmentation (i.e., more intense augmentation during switch than repeat trials and also unattributable to the effects of task rule, within-stimulus incongruence, or preceding erroneous response). We performed this analysis to provide the biological plausibility for direct functional connectivity between distant cortical areas concurrently involved in task switching. It is suggested that cognitive processes are supported by spatially-distinct, simultaneous high-frequency neural events generated by monosynaptically connected cortical networks (Singer, 1993, 2018; Buonomano and Merzenich, 1998; Sonoda et al., 2021).

[Aim 4: Prediction of upcoming responses using a model incorporating high-gamma activity] Finally, we tested the hypothesis that iEEG high-gamma amplitude would predict the accuracy of the forthcoming responses among switch trials. If more intense high-gamma augmentation accurately forecasted higher response accuracy, such neural activation would be considered to support task switching. We hypothesized that enhanced high-gamma amplitude at a region showing *repetition enhancement*, as described in [Aim 2], would facilitate a successful attentional shift to a given updated task rule, and this would increase the chance of responding correctly.

## 2. Methods

### 2.1. Participants

**Fig. 1.**
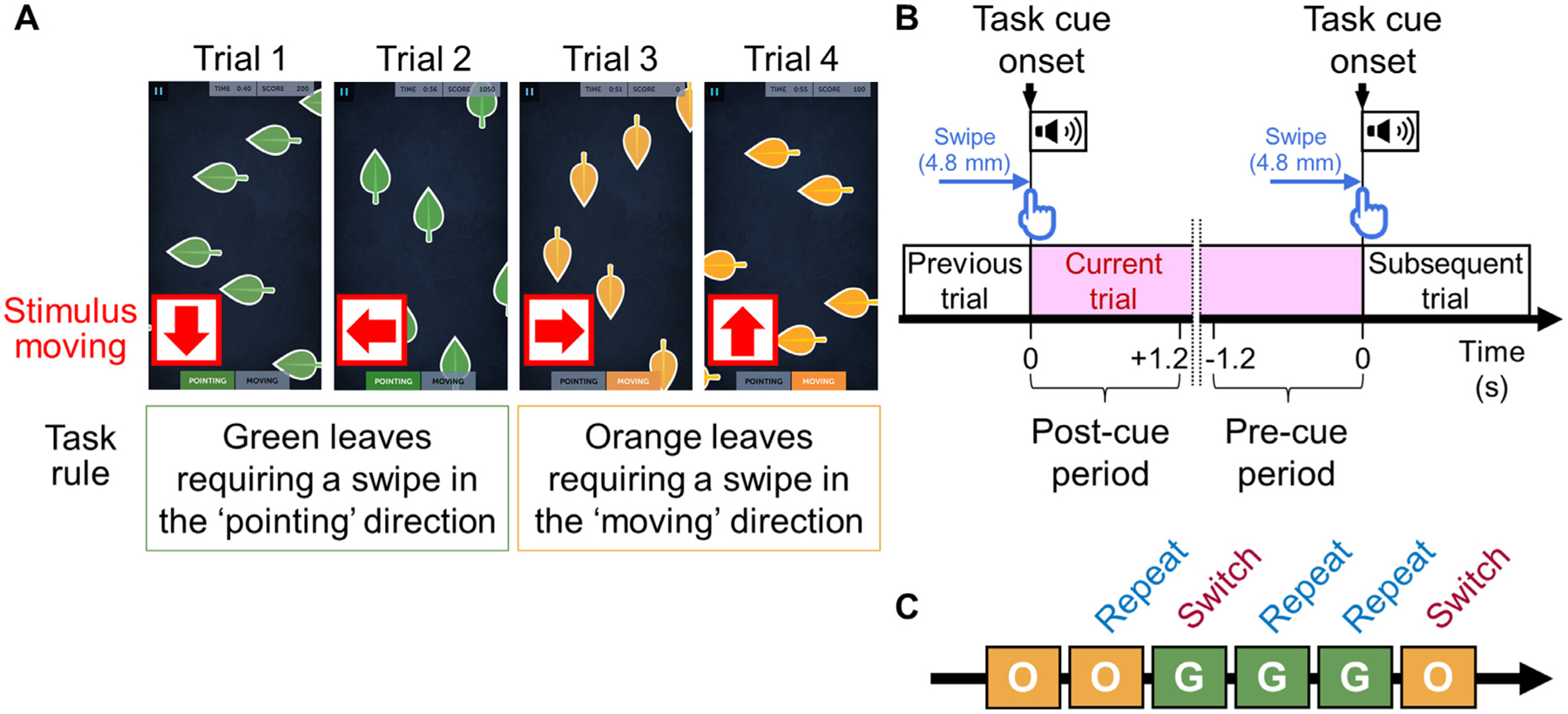
The task-switching paradigm. (A) Examples of task cues in *Ebb and Flow*, a Lumosity cognitive flexibility training game. During Trials 1 and 2, when the leaf color was green, players must swipe the screen in the direction the leaf was pointing (i.e., ‘Left’ on Trial 1 and ‘Up’ on Trial 2). On Trials 3 and 4, when the leaf color was orange, players must swipe in the direction the leaf was moving (i.e., ‘Right’ on Trial 3 and ‘Up on Trial 4 ′). Trial 3 was treated as a switch trial. The task rule (i.e., leaf color) was switched unpredictably; the percentage of same-color run lengths of 1 up to 10 were reportedly 6.4%, 10.6%, 13.5%, 14.8%, 14.3%, 12.6%, 10.0%, 7.3%, 4.8%, and 5.7% (Steyvers et al., 2019). All trials presented here were considered incongruent because the leaves’ pointed direction did not match their moving direction. Video S1 is helpful to understand the paradigm. In each trial, all stimulus leaves moved in a given direction, and none were static. (B) In each trial, a swipe covering 50 pixels (4.8 mm) was detected as a response, which immediately triggered the next cue together with a feedback sound lasting for 0.4–0.6 s. We defined the moment of task cue onset (i.e., response detection = feedback sound onset) as the zero time point in the time-frequency analysis of intracranial electroencephalography signals. With the inherent processing latency of the gameplay platform on our iPad, the screen tapping onset was estimated to be 333 ms, on average, before the zero-time point (95% confidence interval: 325 to 341 ms). We defined a 1200 ms period after the *n* th response as the *n* th post-cue period and a 1200 ms period before the *n + 1* th response as the *n* th pre-cue period (Fig. 3E). (C) A given trial was defined as a switch trial when the leaf color wachanged from orange to green or from green to orange.

The inclusion criteria consisted of (i) extraoperative iEEG recording at Children’s Hospital of Michigan (Detroit, Michigan) between September 2017 and August 2019, (ii) aged four years or older, (iii) played five, 1-minute sessions of *Ebb and Flow* (Fig. 1), a cognitive flexibility game on the Lumosity platform (https://www.lumosity.com/; Lumos Labs, Inc, San Francisco, CA), (iv) gameplay during the interictal state, (v) swipe responses strictly using the same hand, and (vi) correct responses in more than 60% of trials (chance level = 25%). The exclusion criteria included (i) massive brain malformations deforming the central or lateral sulcus (Nakai et al., 2017) and (ii) history of previous resective epilepsy surgery (Nakai et al., 2017). The Institutional Review Ethics Board at Wayne State University has approved the present study. We obtained informed consent from the legal guardians of patients and assent from pediatric patients.

### 2.2. Extraoperative iEEG and MRI data acquisition

**Fig. 2.**
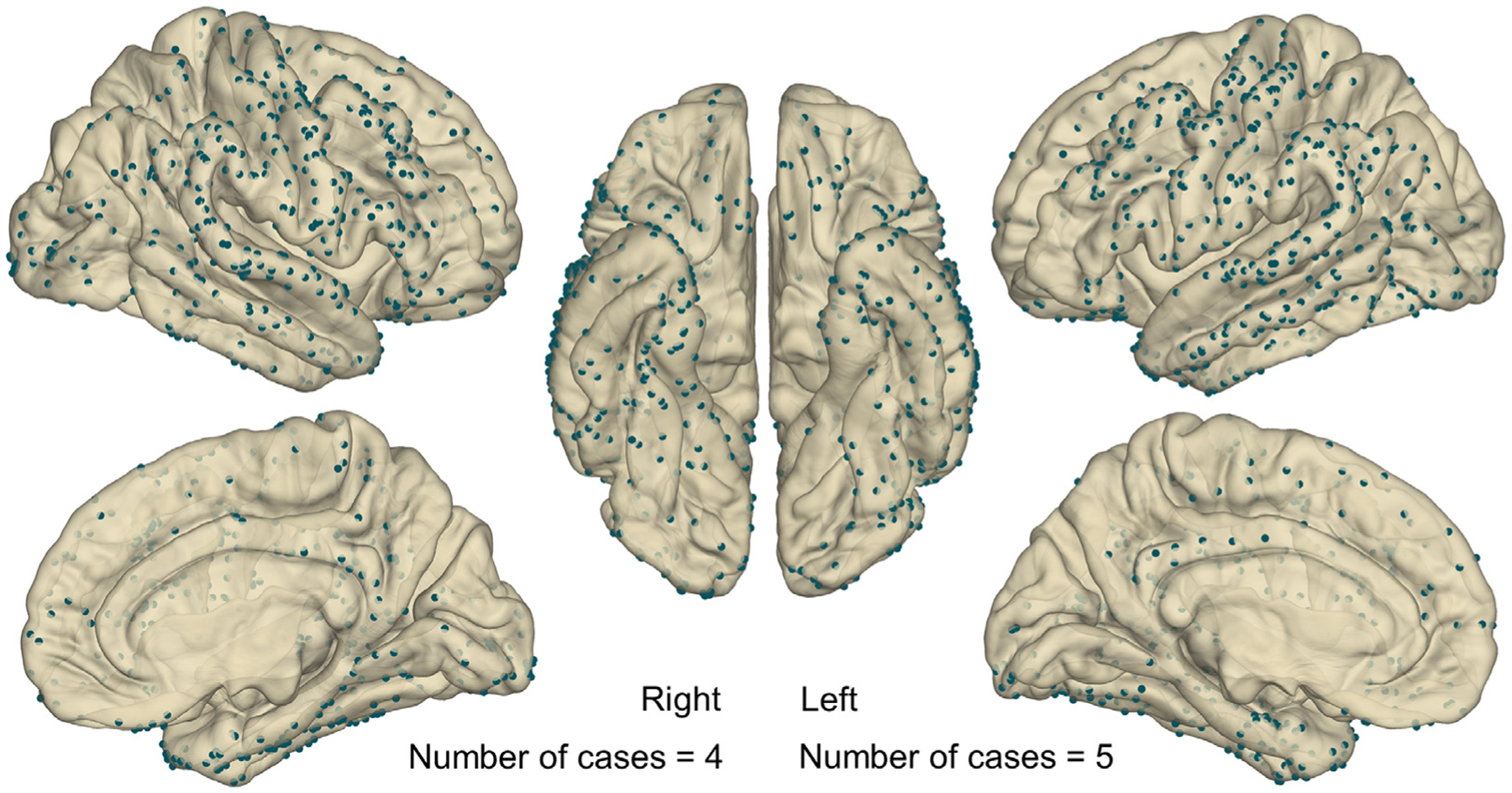
The anatomical locations of subdural electrode contacts included in the analysis. Dark green spheres denote the locations of all electrode sites (four patients had electrode implantation on the right hemisphere; five patients on the left hemisphere). Regions of interest defined in the current study are presented in Fig. S1.

We acquired iEEG and MRI data using methods, as reported previously (Nakai et al., 2017; Mitsuhashi et al., 2021). We placed platinum disk electrodes (10 mm center-to-center distance) on the pial surface of the brain (Fig. 2). Clinical need determined the spatial extent of iEEG sampling, in each patient, with no attempt to place electrodes unnecessary for diagnosis (Asano et al., 2009; Kambara et al., 2018). The sampling rate was 1000 Hz, and the amplifier bandpass was 0.016–300 Hz. We excluded the following electrode sites from further analysis, including the common average reference calculation: those located in the seizure onset zone (Asano et al., 2009), in structural lesions, and those affected by artifacts (Sperling, 2003; Kahane and Dubeau, 2014).

Before intracranial electrode placement, we acquired 3-tesla MRI, including T1-weighted spoiled gradient-recalled echo and fluid-attenuated inversion recovery sequences (Nakai et al., 2017). Using a brain CT scan following electrode implantation, we generated a three-dimensional surface image with implanted electrodes co-registered to anatomically accurate sites on the pial surface (Nakai et al., 2017; Stolk et al., 2018). The FreeSurfer script (http://surfer.nmr.mgh.harvard.edu) spatially normalized given electrode sites to standard Montreal Neurological Institute (MNI) space (Fig. 2; Ghosh et al., 2010; Mitsuhashi et al., 2021).

### 2.3. Task-switch paradigm

Each participant played five sessions of *Ebb and Flow*, during extra-operative iEEG recording (Fig. 1; Video S1). Before initiating the first game session, all patients underwent a brief tutorial session provided by this gaming platform, to understand the task rules. Each session lasted one minute, and participants played five consecutively, at their own pace. All participants played the game on an iPad (screen display width: 14.7 cm; length: 19.6 cm; Apple Inc., Cupertino, CA) in a comfortable position at the bedside. In each trial, the screen displayed a set of leaves moving in a specific direction (either left, right, upward, or downward; Video S1). When the leaf color was green, participants needed to swipe the screen in the same direction the leaves were pointing. When the leaf color was orange, they rather swiped in the direction the leaves were moving. A detected response immediately triggered the next cue with a feedback sound (Video S1), and response time was defined as the interval between two consecutive feedback sound onsets. Feedback sounds were continuously integrated into the iEEG acquisition system via the DC input, temporally synchronizing the gaming and neural signals (Kambara et al., 2018). We treated the task cue onset (i.e., the moment of response detection = feedback sound onset) as the zero time point in the time-frequency iEEG analysis described below. Each trial was classified as [a] ‘task switch’ or ‘repeat’, [b] ‘moving (orange leaves)’ or ‘pointing (green leaves)’ based on the task rule, [c] ‘incongruent’ or ‘congruent’ based on the congruency in direction between the moving and pointing of stimulus leaves, and [d] ‘prior incorrect response’ or ‘prior correct response’ based on the response accuracy in the immediately preceding trial. The first author (T.M.) assessed these trial features strictly based on the simultaneous video and audio recordings while blinded to the results of iEEG analysis. When subsets of the trial features mentioned above (e.g., congruency) were difficult to determine due to suboptimal visibility of the iPad monitor, we treated them as missing values in the mixed model analysis described below.

### 2.4. Assessment of the effect of trial type on patients’ responses

Mixed model analysis tested the hypothesis that, compared to repeat, switch trials were independently associated with increased response times in our patient cohort. The dependent variable was the response time, in a given trial. The fixed effect predictors included [a] task switch (1 if switch), [b] task rule (1 if response was required to be congruent with leaf motion; 0 if congruent with direction of leaf pointing), [c] stimulus leaves’ pointing-moving incongruency (1 if incongruent), [d] prior incorrect response (1 if prior incorrect response), [e] log10-transformed trial number in a given game session, and [f] game session (ranging from 1 to 5). We incorporated a log10-transformed trial number in the mixed model because response times were previously reported to reduce logarithmically, as a function of trial number (Steyvers et al., 2019; Steyvers and Schafer, 2020). The random factors included patient and intercept.

The chi-square test determined whether switch, moving, incongruent trials, and those immediately preceded by an incorrect response more frequently resulted in an incorrect response than given counterparts.

### 2.5. Measurement and visualization of event-related high-gamma modulations

**Fig. 3.**
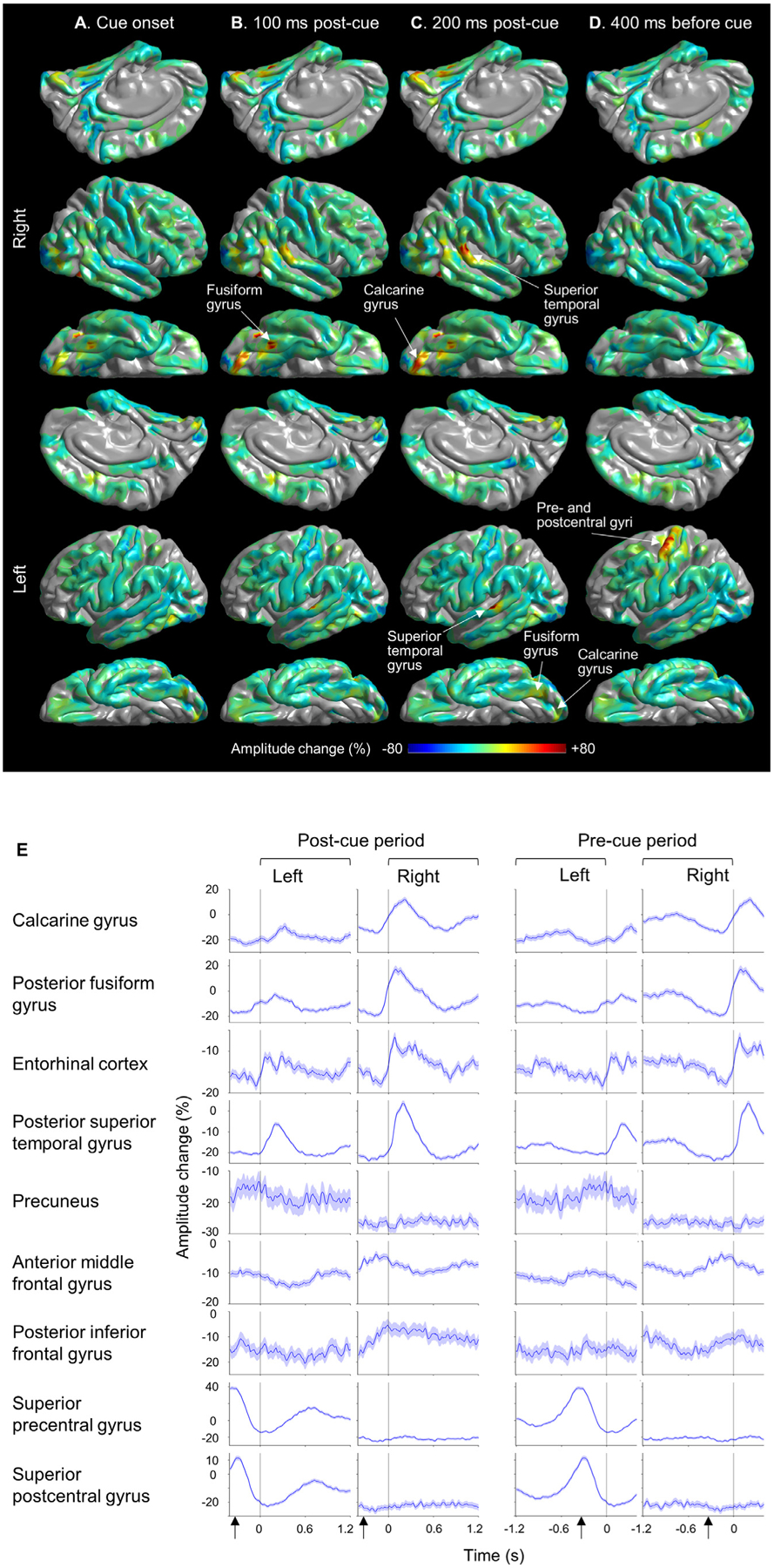
High-gamma modulations during the Lumosity gameplay. (A–D) The snapshots of Video S2 demonstrate the percent change of high-gamma amplitude relative to that during the resting, non-gameplay reference period before the initiation of the Lumosity game. (A) 0 ms: task cue onset (i.e., feedback sound onset/response detection). (B) +100 ms post-cue (C) +200 ms post-cue. (D) 400 ms before task cue onset. (E) The percent change in high-gamma amplitudes at each region of interest (ROI) compared to the non-gameplay reference period. Solid line: mean across all available electrode sites within a given ROI. Blue shade: 95% confidence interval. Arrows: Estimated screen tapping onset (333 ms before the task cue onset). For interested readers, we have provided Fig. S2 showing the broadband evoked responses averaged across all electrode sites within a given ROI.

**Fig. 4.**
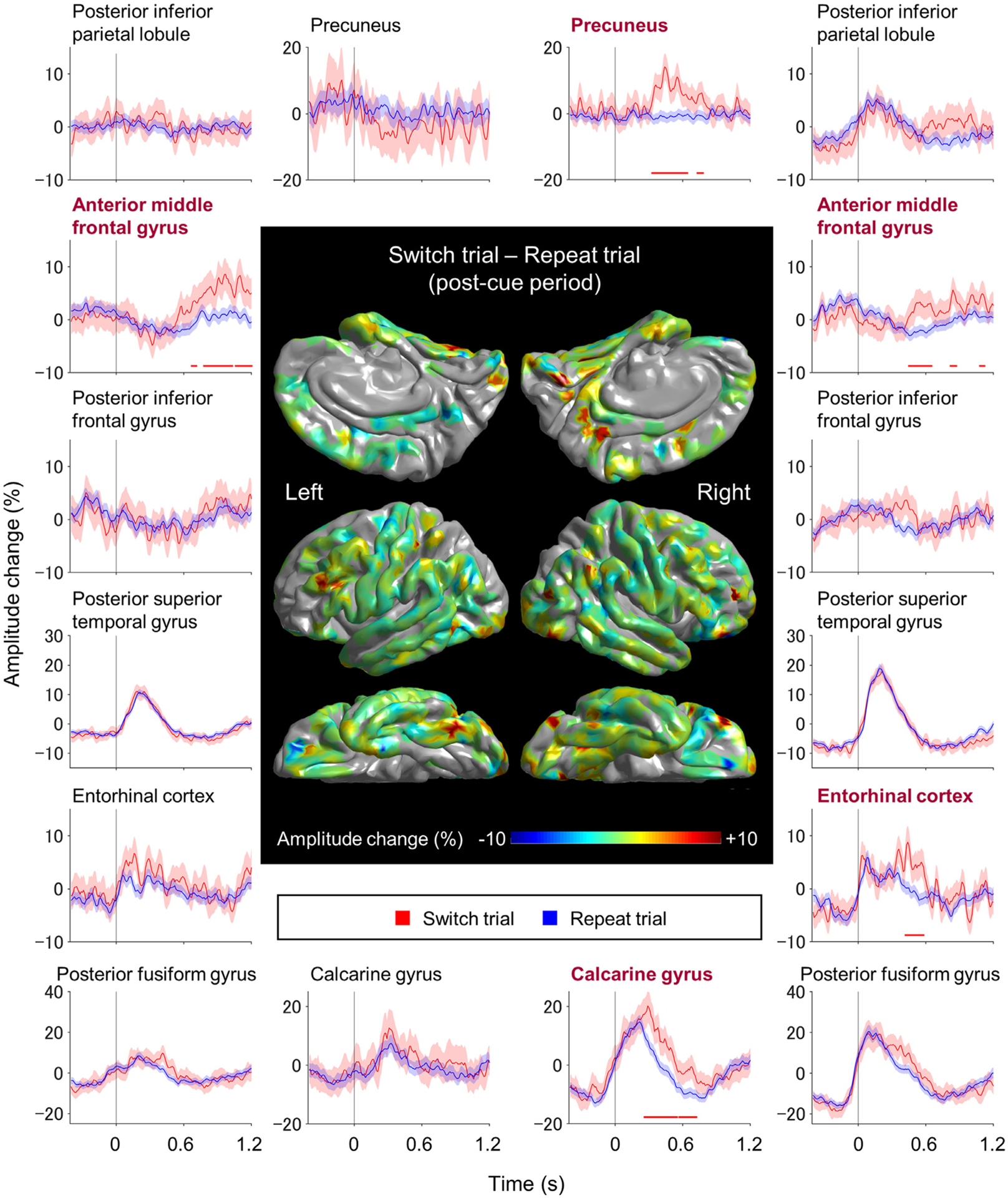
The dynamics of high-gamma modulations in switch and repeat trials. The brain surface map shows the subtraction of high-gamma amplitudes during the 1200 ms post-cue period of repeat trials from those of switch trials. Plots present the dynamics of high-gamma modulations at given regions of interest. Red lines: switch trials. Blue lines: repeat trials. Shading: 95% confidence interval. High-gamma values presented herein are amplitude changes compared to the average during the 1200 ms post-cue period. The zero-time point: task cue onset. With the inherent processing latency of the gameplay platform on our iPad, the screen tapping onset was estimated to be 333 ms before the zero-time point. Horizontal bar: time windows showing a significant difference in high-gamma amplitudes between switch and repeat trials based on the permutation test. Fig. S3 presents the dynamics of high-gamma modulations during the 1200 ms pre-cue period.

**Fig. 5.**
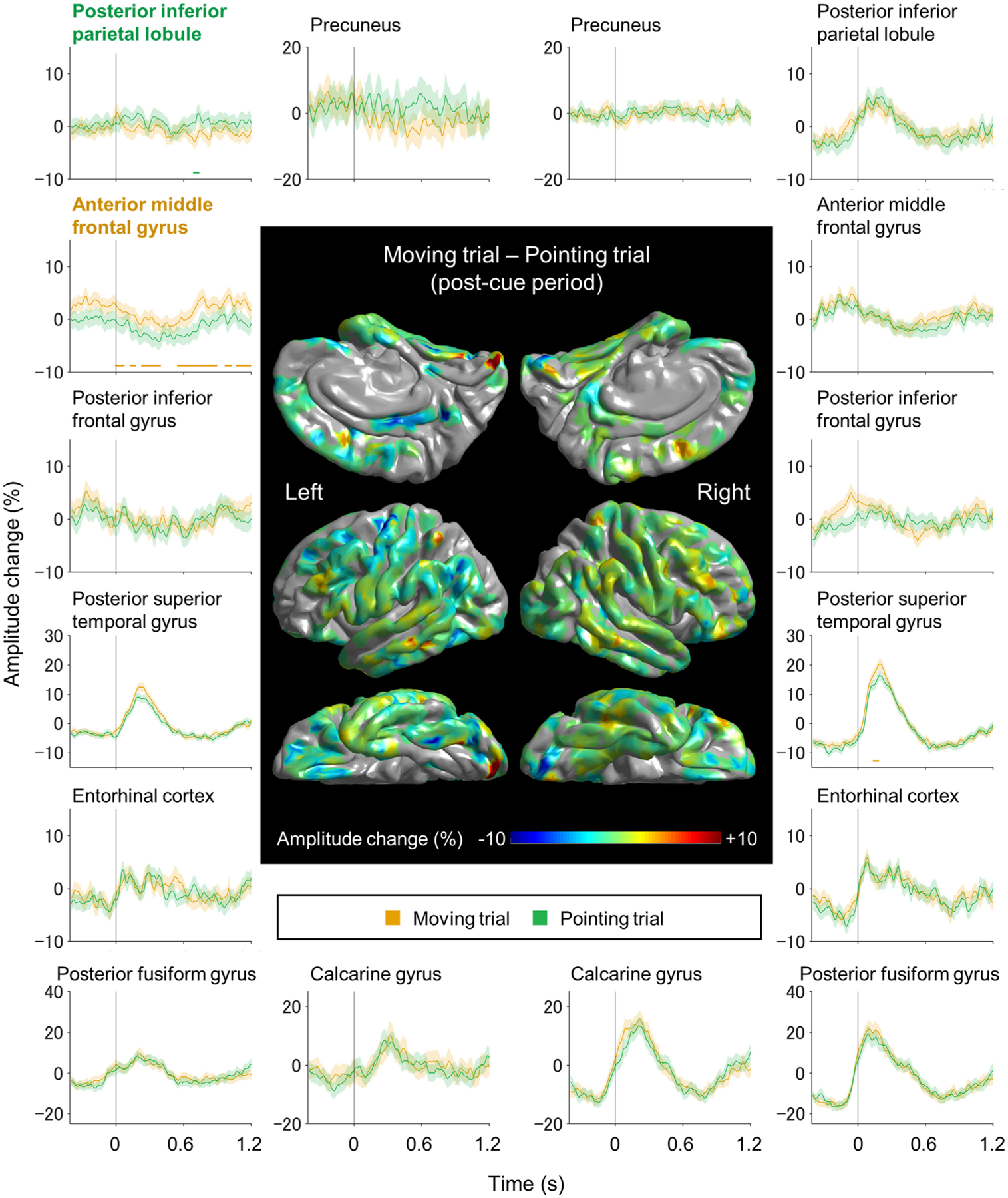
The dynamics of high-gamma modulations in moving and pointing trials. The brain surface map shows the subtraction of high-gamma amplitudes during the 1200 ms post-cue period of pointing trials from those of moving trials. Plots present the dynamics of high-gamma modulations at given regions of interest. Orange lines: moving trials. Green lines: pointing trials. High-gamma values presented herein are amplitude changes compared to the average during the 1200 ms post-cue period. The zero-time point: task cue onset. The screen tapping onset was estimated to be 333 ms before the zero-time point. Horizontal bar: time windows showing a significant difference in high-gamma amplitudes between moving and pointing trials based on the permutation test. Fig. S4 presents the dynamics of high-gamma modulations during the 1200 ms pre-cue period.

**Fig. 6.**
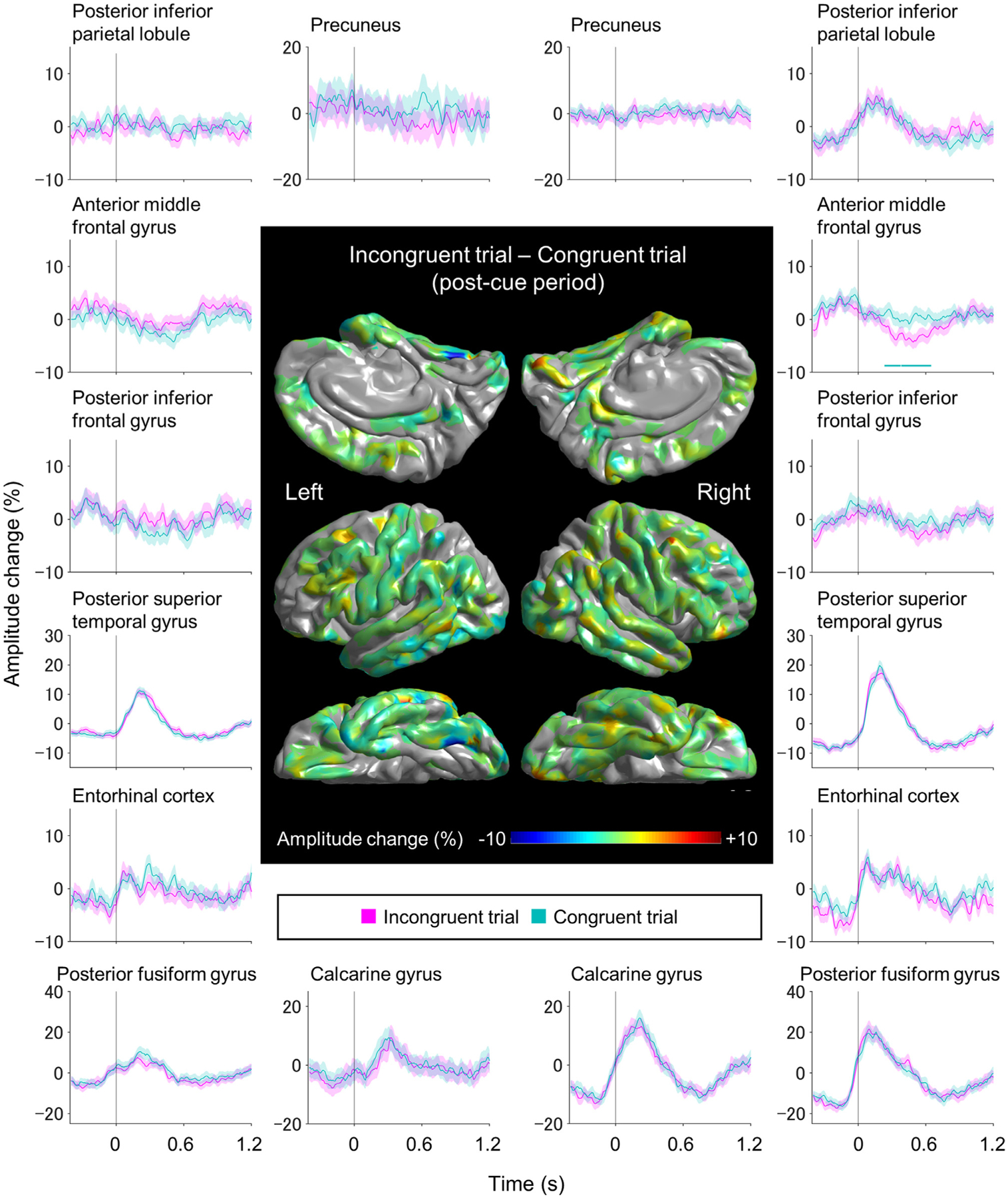
The dynamics of high-gamma modulations in incongruent and congruent trials. The brain surface map shows the subtraction of high-gamma amplitudes during the 1200 ms post-cue period of congruent trials from those of incongruent trials. Plots present the dynamics of high-gamma modulations at given regions of interest. Magenta lines: incongruent trials. Cyan lines: congruent trials. High-gamma values presented herein are amplitude changes compared to the average during the 1200 ms post-cue period. The zero-time point: task cue onset. The screen tapping onset was estimated to be 333 ms before the zero-time point. Horizontal bar: time windows showing a significant difference in high-gamma amplitudes between incongruent and congruent trials based on the permutation test. Fig. S5 presents the dynamics of high-gamma modulations during the 1200 ms pre-cue period.

**Fig. 7.**
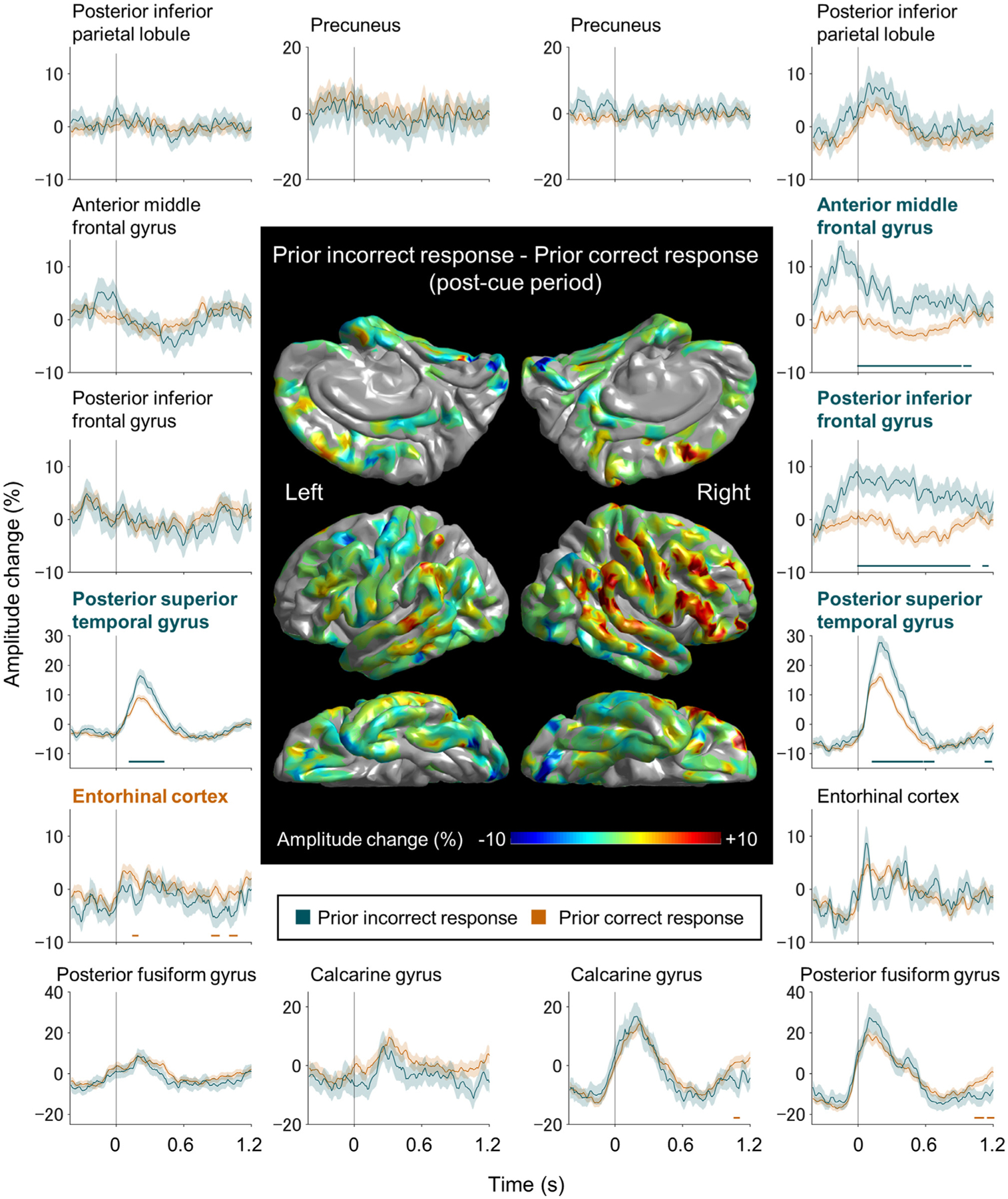
The dynamics of high-gamma modulations in trials preceded by an incorrect and correct response. The brain surface map shows the subtraction of high-gamma amplitudes during the 1200 ms post-cue period of trials preceded by a correct response from those of trials preceded by an incorrect response. Dark green lines: trials preceded by an incorrect response. Brown lines: trials preceded by a correct response. High-gamma values presented herein are amplitude changes compared to the average during the 1200 ms post-cue period. The zero-time point: task cue onset. The screen tapping onset was estimated to be 333 ms before the zero-time point. Horizontal bar: time windows showing a significant difference in high-gamma amplitudes between trials preceded by an incorrect and correct response based on the permutation test. Fig. S6 presents the dynamics of high-gamma modulations during the 1200 ms pre-cue period.

We measured event-related high-gamma activity, as reported in our previous studies (Mitsuhashi et al., 2021; Sonoda et al., 2022). Using the FieldTrip toolbox (http://www.fieldtriptoolbox.org/), we performed the Morlet wavelet time-frequency analysis on iEEG signals referenced to a common average (Crone et al., 2001; Mitsuhashi et al., 2021). We transformed iEEG voltage signals during a 2400 ms period centered on each task cue onset into time-frequency bins, at a 30–110 Hz broadband range (5 Hz frequency bins; a given frequency band divided by seven cycles; sliding in 10 ms steps; Mitsuhashi et al., 2021). To compare gameplay-related high-gamma amplitude (a measure proportional to the square root of high-gamma power) with that during the baseline period before task initiation, we sampled 2400 ms resting wakefulness epochs as many as the total number of trials played by a given patient. To minimize the potential effects of interictal spikes on gameplay-related high-gamma modulations, we excluded time-frequency bins with 30–85 Hz amplitude above an absolute z-score of 2 from further analysis (Sonoda et al., 2022). Next, we computed the percent change of high-gamma (70–110 Hz) amplitude at each electrode site, compared to the *non-gameplay* reference mean (Fig. 3; Video S2). We also calculated and visualized the high-gamma amplitude changes relative to the mean of each *gameplay* period (Figs. 4–7; Video S3). Finally, we created movies animating the spatiotemporal dynamics of high-gamma amplitude changes on the average FreeSurfer pial surface image with a Gaussian half-width at half-maximum of 7.5 mm (Videos S2 and S3).

### 2.6. [Aim 1] region of interest (ROI) analyses of event-related high-gamma modulations

We presented plots to visualize the time windows in which high-gamma amplitudes were modulated (i.e., augmented or suppressed), compared to the non-gameplay reference period (Fig. 3E). A nonparametric permutation one-sample *t*-test evaluated the null hypothesis that, compared to the reference period, the high gamma amplitude percent change would be zero at each 10-ms bin (1000 permutations; random sign swapping; Maris and Oostenveld, 2007; Cohen, 2014; Bassez et al., 2020; Sonoda et al., 2022). A two-sided 5% significance level was used with a false discovery rate (FDR) correction for repeated comparisons for 121 bins in a 2400 ms period (Fig. 1B).

We also provided plots visualizing the effects of [a] task switch (Fig. 4), [b] task rule (Fig. 5), [c] stimulus incongruency (Fig. 6), and [d] prior incorrect response (Fig. 7) on the gameplay-related high-gamma amplitudes, at a given region of interest (ROI) defined by the Desikan FreeSurfer Atlas (Fig. S1; Desikan et al., 2006; Nakai et al., 2017). The permutation test likewise determined the time windows showing significant differences in high-gamma amplitudes between two given trial types. We treated high-gamma augmentation as “trial type-specific”, when it showed significantly greater high-gamma amplitudes during a particular trial type (e.g., switch), based on both the permutation test mentioned above and the following mixed model analysis. The purpose of this mixed model analysis was to determine whether the observed difference in high-gamma amplitudes between trial types (e.g., switch vs. repeat) was unattributable to the effects of the other trial domains (e.g., task rule, stimulus incongruency, or prior incorrect response). The dependent variable in this mixed model analysis was high-gamma amplitudes during a given trial at each ROI. The fixed effect predictors included [a] task switch, [b] task rule, [c] stimulus incongruency, [d] prior incorrect response, [e] log10-transformed trial number, and [f] game session. The random factors included patient and intercept. In this mixed model analysis, we performed an FDR correction for repeated analyses for 56 ROIs that included electrode sites (Fig. S1).

[Aim 2] The aforementioned mixed model analysis was designed to clarify the association between practice-related behavioral improvement and local neural activity. If a mixed model estimate was smaller than zero for log10-transformed trial number or game session, a given ROI was considered to show repetition suppression. In contrast, a mixed model estimate larger than zero would suggest repetition enhancement. The mixed model analysis furthermore determined whether high-gamma amplitudes were likewise reduced through practice at the whole-electrode level.

### 2.7. [Aim 3] structural connectivity between cortical regions showing switch-related high-gamma augmentation

The DWI tractography analysis determined whether direct white matter tracts existed between distant ROIs showing switch-related high-gamma augmentation with temporal proximity (i.e., not more than 50 ms apart). Investigators have suggested that large-scale cortico-cortical neural propagations through direct white matter tracts generally take 50 ms or less (Alarcon et al., 1994; Matsumoto et al., 2017). As previously performed (Mitsuhashi et al., 2021; Sonoda et al., 2021), we generated DWI tractography using the open-source data averaged across 1065 individuals participating in the Human Connectome Project (http://brain.labsolver.org/diffusion-mri-templates/hcp-842-hcp-1021; Yeh et al., 2018). We previously validated this analytic approach by demonstrating that neural propagations based on the open-source data were spatially similar to those found on the individual DWI (Sonoda et al., 2021). We placed seed-spheres with a radius of 4 mm at all electrode sites within ROIs with switch-related high-gamma augmentation. The DSI Studio (http://dsi-studio.labsolver.org/) generated streamlines between these ROIs within Montreal Neurological Institute (MNI) standard space. We built a connectome map based on the number of streamlines for given edges and a 3D map of the observed streamlines. We considered streamlines satisfying the following criteria to be legitimately significant: a fractional anisotropy threshold of 0.5, a maximum turning angle of 70°, a step size of 0.3 mm, and a streamline length of 10 to 250 mm.

### 2.8. [Aim 4] prediction of upcoming responses using iEEG high-gamma activity

Using trial-by-trial iEEG analysis, we tested the hypothesis that successful high-gamma augmentation at an ROI showing repetition enhancement, as revealed in [Aim 2], would help optimize response accuracy during switch trials. A multivariate logistic regression-based model classified the response accuracy (1 if an upcoming response was correct). The predictors included [a] high-gamma amplitude (% change) in a given trial, [b] task rule, [c] stimulus incongruency, and [d] game session. This logistic regression-based prediction model did not incorporate behavioral variables such as a prior incorrect response or log10-transformed trial number in each 1 min game session, to minimize the risk of circular analysis (Kriegeskorte et al., 2009). A receiver operating characteristics (ROC) analysis (Kuroda et al., 2021) determined how accurately the logistic regression model incorporating high-gamma activity classified the upcoming responses during switch trials. We employed a 5-fold cross-validation approach to reduce the risk of over-fitting; in short, one trial was excluded from the initial logistic regression model and used to assess its performance. A total of 1209 trials were available for this analysis.

### 2.9. Statistical analysis

Statistical analyses were performed using ‘IBM SPSS Statistics version 27′ (IBM Corp., Armonk, NY, USA) and ‘Statistical and Machine Learning Toolbox MATLAB 2018b’ (Mathworks, Natick, MA, USA). The significance was set at an FDR *p*-value of 0.05.

### 2.10. Data and code availability

All iEEG data and the MATLAB-based codes used in the analyses are available upon request to the corresponding author.

## 3. Results

### 3.1. Behavioral observations

**Fig. 8.**
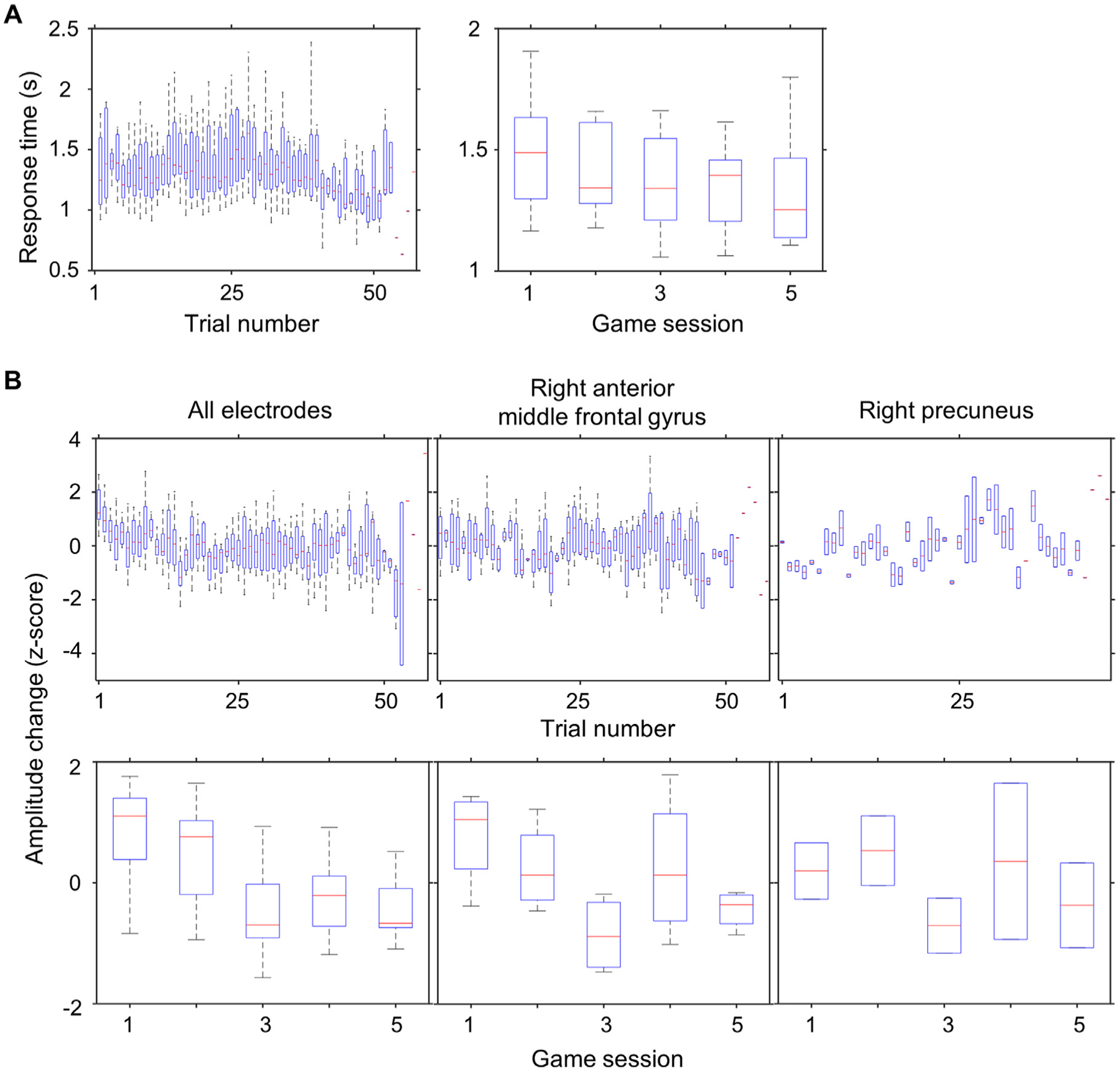
Changes in response time and gameplay-related high-gamma amplitudes with practice. (A) Changes in response time are presented. Left: as a function of trial in a given session. Right: as a function of game session. (B) Changes in high-gamma amplitudes are presented. Left: all 860 electrode sites. Middle: right anterior middle-frontal region (29 sites). Right: right precuneus region (6 sites).

Nine patients who satisfied the inclusion and exclusion criteria (Table 1) were investigated. We analyzed an average of 95.6 [standard deviation: ±16.9] subdural electrodes from each patient (Fig. 2). The total number of trials per patient was 221.4 [±33.7], which corresponded to 37.3 [±5.8] switch, 111.1 [±19.9] moving, and 103.7 [±19.8] incongruent, and 49.7 [±16.0] trials were immediately preceded by an incorrect response. The mean response time was 1.36 [±0.52] seconds. Fig 8 shows changes in response time, as a function of trial in a given session and game session.

Mixed model analysis of trial type demonstrated that switch, moving, incongruent trials, and those immediately preceded by an incorrect response independently increased the response times (Table 2). In contrast, a later game session was independently associated with reduced response times (Table 2). The mean proportion of correct responses was 77.6 [±5.6]%, and the proportion of incorrect responses was significantly higher, compared to complimentary trial types, in switch, moving, and incongruent trials, as well as those immediately preceded by an incorrect response (Table 3).

### 3.2. Animation of neural modulations during gameplay

Videos S2 and S3 demonstrate the spatiotemporal dynamics of high-gamma modulations during *Ebb and Flow* gameplay. Compared to the non-gameplay resting period, ROI-based time-frequency analysis revealed high-gamma amplitude augmentation maximally between +80 and +330 ms post-cue, in visual pathways including the calcarine, fusiform, and entorhinal gyri of each hemisphere (Fig. 3B). High-gamma amplitudes were likewise increased in the posterior superior-temporal gyrus (STG) of both hemispheres, peaking at +210 and +190 ms post-cue (Fig. 3C). In the left precentral and postcentral gyri, high-gamma amplitudes were enhanced most drastically at 380 and 290 ms before task cue onset, respectively (i.e., at 47 ms before and 43 ms after the estimated onset of screen tapping; Fig. 3D and E).

### 3.3. High-gamma augmentation specific to switch trials

[Aim 1] The ROI-based iEEG analysis identified the spatiotemporal characteristics of high-gamma augmentation specific to switch trials. Fig. 4 contrasts the difference in high-gamma amplitudes between switch and repeat trials, at each ROI. Specifically, the permutation test demonstrated that the right calcarine, right precuneus, right entorhinal, and bilateral anterior middle frontal gyrus (MFG) regions showed high-gamma amplitudes greater during switch trials, compared to repeat ones. According to the permutation test, significant difference was noted in the right calcarine between +260 and +720 ms post-cue (maximum difference: 12.6% at +310 ms), right precuneus between +330 and +780 ms post-cue (maximum difference: 15.2% at +440 ms), right entorhinal between +420 and +580 ms post-cue (maximum difference: 8.6% at +440 ms), right anterior MFG between +450 and +1200 ms post-cue (maximum difference: 5.9% at +840 ms post-cue), and left anterior MFG between +670 and +1200 ms post-cue (maximum difference: 8.3% at +1100 ms post-cue). At each of these five ROIs, mixed model analysis demonstrated that switch trials were associated with increased high-gamma amplitudes compared to repeat trials (*p* < 0.001 and *t* = +4.271 at right calcarine; *p* < 0.001 and *t* = +5.247 at right precuneus; *p* = 0.008 and *t* = +3.445 at right entorhinal; *p* = 0.013 and *t* = +3.117 at right anterior MFG; *p* < 0.001 and *t* = +5.134 at left anterior MFG), independently of the effects of task cue, stimulus incongruency, and immediately prior response.

[Aim 2] Fig. 8 demonstrates the changes in high-gamma amplitude with practice. The mixed model analysis, employed at all 860 electrode sites, demonstrated that an increase in the log10-transformed trial number within a given game session (mixed model estimate: −1.35; *p* < 0.001; *t* = −14.700) and a later game session (mixed model estimate: −0.30; *p* < 0.001; *t* = −12.154) independently reduced overall post-cue high-gamma amplitudes.

The ROI-based mixed model analysis (Tables S1–S5) likewise demonstrated that an increase in the log10-transformed trial number reduced the post-cue high-gamma amplitudes in the right calcarine (mixed model estimate: −5.63; *p* < 0.001; *t* = −5.845) and right anterior MFG (mixed model estimate: −1.91; *p* < 0.001; *t* = −3.664). Additionally, a later game session was associated with reduced post-cue high-gamma amplitudes in the right calcarine (mixed model estimate: −0.33; *p* = 0.047; *t* = −2.481), right entorhinal (mixed model estimate: −0.73; *p* < 0.001; *t* = −6.704), right anterior MFG (mixed model estimate: −0.71; *p* < 0.001; *t* = −4.988), and left anterior MFG (mixed model estimate: −0.58; *p* < 0.001; *t* = −3.783).

Conversely, an increase in the log10-transformed trial number was found to increase the post-cue high-gamma amplitudes in the right precuneus (mixed model estimate: +2.2; *p* = 0.003; *t* = +2.958).

### 3.4. High-gamma augmentation specific to moving trials

The ROI-based analysis identified the spatiotemporal characteristics of high-gamma augmentation specific to moving trials. Fig. 5 contrasts the difference in high-gamma amplitudes between moving and pointing trials, at each ROI. The permutation test demonstrated that moving trials enhanced and sustained high-gamma amplitude at the left anterior MFG region, and this augmentation was statistically significant, independent of the remaining fixed-effect predictors (between +0 and +1200 ms post-cue; maximum difference: 5.2% at +990 ms post-cue; *p* = 0.038; *t* = +2.646).

### 3.5. High-gamma augmentation specific to incongruent trials

The ROI-based analysis failed to identify ROIs showing high-gamma modulations specific to incongruent trials. Fig. 6 contrasts the difference in high-gamma amplitudes between incongruent and congruent trials at each ROI. The permutation test demonstrated that the right anterior MFG region showed high-gamma amplitudes smaller during incongruent trials (between +240 and +640 ms post-cue; maximum difference: −5.5 % at +510 ms post-cue). However, the mixed model analysis failed to demonstrate the effect of stimulus incongruency was independent of the remaining fixed-effect predictors (*p* = 0.835; *t* = −0.508).

### 3.6. High-gamma augmentation specific to trials preceded by incorrect responses

The ROI-based analysis identified the differing spatiotemporal characteristics of high-gamma augmentation specific to trials immediately preceded by an incorrect or correct response, and the results are displayed in Fig. 7. Significant differences were noted in the right inferior-frontal gyrus (IFG) region between +0 and +1150 ms post-cue (maximum difference: 11.9% at +490 ms), right anterior MFG between +0 and +1000 ms post-cue (maximum difference: 9.2% at +130 ms), right posterior STG between +130 and +670 ms post-cue (maximum difference: 13.4% at +260 ms), and left posterior STG between +120 and +420 ms post-cue (maximum difference: 7.8% at +220 ms). At each of these four ROIs, the mixed model analysis demonstrated that trials preceded by an incorrect response were associated with increased high-gamma amplitudes (*p* < 0.001 and *t* = +12.647 at right IFG; *p* < 0.001 and *t* = +7.864 at right anterior MFG; *p* < 0.001 and *t* = +6.484 at right STG; *p* < 0.001 and *t* = +5.566 at left STG), independent from the effects of task switch, task rule, and stimulus incongruency.

### 3.7. [Aim 3] structural connectivity between task switch-related cortical regions

**Fig. 9.**
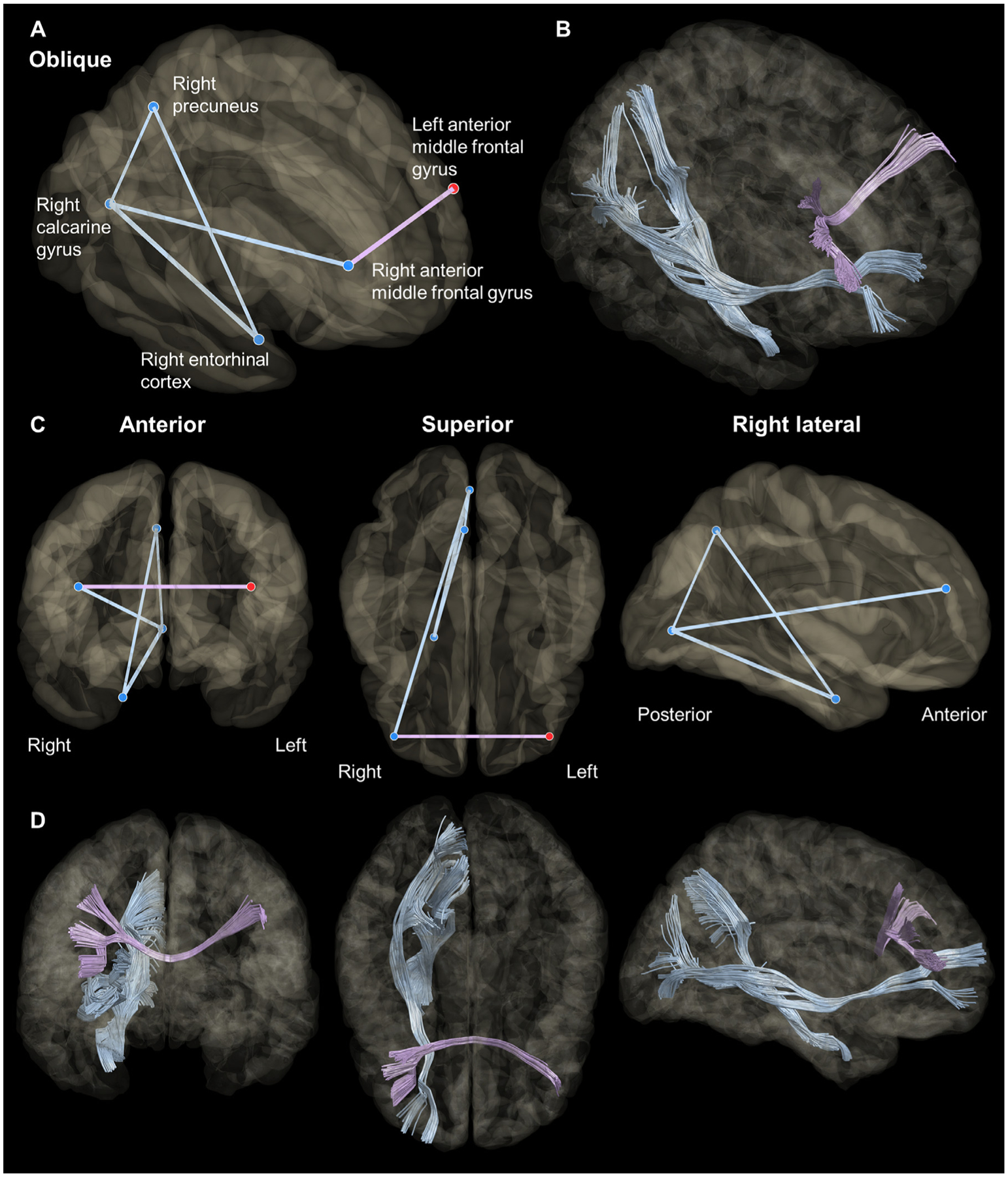
Structural connectivity between cortical regions specifically involved in task switching. (A and C) show the connectome between the cortical regions showing switch-related high-gamma augmentation. The blue and red spheres represent nodes in the right and left hemispheres, respectively. The light blue and purple lines represent the edges within the right hemisphere and across the hemispheres, respectively. Thick lines reflect greater numbers of streamlines identified on diffusion-weighted imaging tractography. (B and D) show the anatomical courses of given streamlines.

Fig. 9 demonstrates the structural connectome between cortical areas showing switch-related high-gamma augmentation. Direct white matter tracts were found from the right calcarine to precuneus (number of streamlines: 87), entorhinal (1157), and anterior-middle frontal regions (603), between the right entorhinal and precuneus regions (211), as well as connecting the right and left anterior MFG regions (1025).

### 3.8. [Aim 4] high gamma-based prediction of upcoming responses among switch trials

We performed this analysis on the right precuneus ROI, which showed switch-related high-gamma augmentation during +330 to +780 ms post-cue period (Fig. 4) as well as enhancement in high-gamma responses as a function of the log10-transformed trial number (Fig. 8). The multivariate logistic regression analysis indicated that right precuneus high-gamma amplitude independently had a modest, yet significant impact on the accuracy of predicting responses among switch trials. Each 1% increase in the right precuneus high-gamma amplitude increased the odds of responding correctly by 1.003 times (i.e., *e*^1.003;^ 95%CI: 1.000 to 1.007). Furthermore, progressing forward by one session also increased the odds of correct responses by 1.121 (95%CI: 1.048 to 1.193). The logistic regression model’s accuracy was calculated via ROC analysis, and it indicated that correct responses were predicted with an area under the curve of 0.674 (95%CI: 0.563 to 0.785).

## 4. Discussion

### 4.1. Temporally and functionally distinct large-scale brain network dynamics involved in task switching

To our knowledge, this is the first-ever study that identified temporally, functionally distinct large-scale cortico-cortical network dynamics that support task switching. We refer to one as the *visual-precuneus network* and the other as the *visual-dorsolateral prefrontal network*. Based on the current study results, both networks are believed to originate from the low- and high-order ventral visual areas, including the calcarine and entorhinal regions. The visual-precuneus network is proposed to include the precuneus, whereas the visual-dorsolateral prefrontal network includes the anterior MFG.

Our findings suggest that the visual-precuneus network is engaged in task switching before the dorsolateral prefrontal region. Our iEEG analysis revealed that switch-related high-gamma augmentation in the visual and precuneus areas occurred at least 120 ms before that in the anterior MFG region. The networks’ anatomical feasibility was confirmed by our DWI tractography analysis, as it revealed direct white matter connectivity from the visual to the precuneus and anterior MFG in the right hemisphere; interestingly, it failed to show connectivity between the precuneus and anterior MFG.

Synthesizing the above, we propose that the visual-precuneus and visual-dorsolateral networks play distinct, functional roles, given their distinct, local high-gamma responses as a function of trials. The right precuneus showed repetition enhancement, whereas the right anterior MFG showed repetition suppression similar to the overall brain regions.

### 4.2. Roles of the visual-precuneus network dynamics in task switching

The visual-precuneus network dynamics may be involved in awareness of task switching cues and allocating attention to the newly switched cues, considering the timing of high-gamma augmentation and the findings reported in previous literature. The collective evidence from previous lesion, electrophysiology, fMRI, and stimulation studies suggests that the ventral visual pathways are essential for perceiving and recognizing changes in shape, color, and movement of objects (Zeki 1990; Riesenhuber and Poggio, 2000; Gilaie-Dotan et al., 2013; Kravitz et al., 2013; Lafer-Sousa et al., 2016). The existence of direct connectivity on DWI tractography (Fig. 8), concurrent iEEG high-gamma augmentation related to task switching (Fig. 4), and functional connectivity on resting-state fMRI in healthy adults (Zhang and Li, 2012) support the notion that the precuneus receives neural input directly from visual areas. Previous fMRI studies of healthy adults indicate that tasks requiring a shift of visual attention were associated with increased hemodynamic responses in the precuneus regions (Shulman et al., 2009; Huijbers et al., 2011; Tosoni et al., 2013), while conversely, lesions involving the precuneus impaired this process (Vandenberghe and Gillebert, 2009).

The observed repetition enhancement of high-gamma amplitudes in the right precuneus is consistent with the notion that precuneus activations trigger an attentional shift during switch trials. Our iEEG analysis among switch trials indicated that each 1% elevation of precuneus high-gamma amplitude increased the chance of responding correctly to the upcoming stimulus by approximately 0.3%. Further studies using electrical stimulation mapping may be warranted to determine whether neural activation in the right precuneus region is essential for task switching.

### 4.3. Roles of the visual-dorsolateral prefrontal network dynamics in task switching

The visual-dorsolateral prefrontal network dynamics may be involved in executing appropriate stimulus-response translation in switch trials without interference from old rules (Brass et al., 2003; Koechlin et al., 2003; Sohn et al., 2007; Hyafil et al., 2009; Nee and D’Esposito, 2017). To the point, many fMRI studies of healthy adults reported that more intense hemodynamic activations were induced in the dorsolateral prefrontal regions when a task switch was required (Dove et al., 2000; Sohn et al., 2000; Braver et al., 2003; Badre and Wagner, 2006; Yeung et al., 2006; Karayanidis et al., 2010; Kim et al., 2012; Jamadar et al., 2015; Vallesi et al., 2015; Cole et al., 2016). The current study provided anatomical justification for this phenomenon, demonstrating that the inferior fronto-occipital fasciculus directly connects the right visual and anterior MFG regions (Fig. 9). In this present work, switch-related high-gamma augmentation co-occurred in the right visual and anterior MFG regions (Fig. 4). A previous iEEG study using single-pulse electrical stimulation provided evidence that low-order visual areas can directly transfer neural information to the dorsolateral prefrontal regions within 50 ms (Sugiura et al., 2020). Thus, it is plausible to expect the right anterior MFG could receive neural input directly from the visual areas.

High-gamma amplitudes in the right anterior MFG showed repetition suppression, based on our trial-by-trial iEEG time-frequency analysis. Such reduction of overall high-gamma amplitudes, including those seen in the visual-dorsolateral prefrontal network, likely reflects a practice-related decrease of neural cost related to task switching.

### 4.4. Network dynamics involved in tracking moving objects

The present study also uncovered the spatiotemporal dynamics of high-gamma modulations related to functions other than task switching. For example, higher behavioral and neural costs were required for swiping in the direction of leaf motion, compared to leaf pointing. In this vein, moving trials were associated with *a* > 100 ms longer response time, about 20% lower response accuracy, and continuously greater high-gamma amplitudes in the bilateral anterior MFG (Fig. 5). A possible explanation is that movement recognition required more laborious cortical processing than shape recognition. During pointing trials, a single glance may be sufficient to identify leaf orientation and make a relevant response. Conversely, additional spatial working memory may be required during moving trials to mentally store the position of leaves and then recognize their directional movement. Lesion studies infer that the dorsolateral prefrontal regions, including the anterior MFG, are necessary for spatial working memory in humans (Barbey et al., 2013; Mackey et al., 2016). Non-human primate analogues of these studies suggest that the dorsolateral prefrontal regions are involved in transient coding of visual space (Funahashi et al., 1989; Kim and Shadlen, 1999), lending support to our hypothesis.

### 4.5. Network dynamics occurring after erroneous responses

Post-error high-gamma augmentation appeared in the right IFG and anterior MFG, at the moment of response detection (Fig. 7). Both IFG and MFG post-error high-gamma augmentation preceded that in the bilateral STG by > 100 ms (Fig. 7). Thus, it is plausible to consider that at least certain patient subsets may have realized their erroneous responses before receiving feedback sounds. Trials immediately preceded by an error were associated with *a* > 150 ms longer response time and about 25% lower response accuracy. Post-error response high-gamma augmentation in the right IFG and anterior MFG may reflect the neural processes for detecting incorrect responses and subsequent behavioral adjustment to prevent future transgressions. Similarly, previous fMRI and iEEG studies reported that erroneous responses during spatial-attention tasks requiring a choice among competing options were associated with increased hemodynamic and high-gamma activations in the IFG and MFG with right-hemispheric dominance (Huster et al., 2011; Rae et al., 2014; Völker et al., 2018). A previous iEEG case study reported that high-gamma augmentation took place in the right IFG when preparing to stop in a stop-signal task (Swann et al., 2012), and lesion studies have localized such inhibitory function to the right IFG (Aron et al., 2004).

### 4.6. Usage of computer tablet game in functional brain mapping

The present study has provided preliminary evidence that time-frequency iEEG analysis during gameplay on a tablet computer can help localize eloquent cortices to be preserved in brain surgery. This finding has the potential to drastically improve extra-operative mapping for pediatric focal epilepsy patients, since electrical stimulation mapping is not always efficacious in young children (Haseeb et al., 2007) and was reported to localize language areas in less than 20% of those younger than ten years old (Schevon et al., 2007). Measurement of event-related high-gamma modulations is widely practiced in tertiary epilepsy surgery centers but previously reported tasks may not have been entertaining for children (Crone et al., 2011; Lachaux et al., 2012; Arya et al., 2018; Mooij et al., 2016; Sonoda et al., 2022). In our current study, patients made an average of 221.4 responses during five-minute gameplay sessions. The task cue onset (accompanied by feedback sounds) induced high-gamma augmentation in the low- and high-order visual areas as well as in the posterior STG regions bilaterally (Fig. 3B, C). Apart from the precuneus and MFG, swipe responses using the right finger induced high-gamma augmentation in the left pre- and post-central gyri (Fig. 3D). Real-time mapping tools (Brunner et al., 2009; Korostenskaja et al., 2014; Wang et al., 2016) is expected to improve the practicality of using task-related high-gamma modulations in epilepsy presurgical evaluation.

The present study successfully localized swipe-related neural activation in the primary sensorimotor area contralateral to the hand used for responses. Since inception of the iPhones in 2007, these finger gestures are an evolutionary new behavior that humans have rapidly mastered over the past 14 years, and now 3.8 billion people worldwide are estimated to own a smartphone, as of July 2021 (https://www.bankmycell.com/blog/how-many-phones-are-in-the-world).

### 4.7. Methodological considerations

In *Ebb and Flow*, a task cue (i.e., green or orange leaves) and a target stimulus (i.e., pointing and moving leaves) are presented simultaneously; thus, the cue-target interval was effectively zero in the present study. Conversely, in scalp EEG studies, investigators designed tasks with several hundred-millisecond cue-target intervals (Nicholson et al., 2005; Jamadar et al., 2010; Karayanidis and Jamadar, 2014). Such cue-target intervals allowed differential ERP measurements [a] during a proactive (anticipatory) period immediately after presenting a cue indicating a given task rule and [b] during a reactive period after target presentation. Switch trial-preferential ERPs on scalp recording were characterized by a surface-positive deflection in the posterior brain region during a proactive period and a deflection with a frontal-positive/posterior-negative dipole during a reactive period (Jamadar et al., 2010). Some may hypothesize that switch-related high-gamma augmentations observed in the present study reflect a mixture of neural processes reported to occur differentially during proactive and reactive periods (Karayanidis and Jamadar, 2014).

Limited iEEG signal sampling, size of ROIs, and number of iEEG frequency bands analyzed in the present study may account for the failure to find neural engagement more intense in incongruent than congruent trials. One cannot rule out the possibility that iEEG electrodes failed to sample critical sites or that a given ROI may have been too large to include critical sites specifically. For example, the medial frontal region, such as the anterior cingulate cortex, was sparsely sampled in the present study, and this region reportedly shows intense high-gamma and hemodynamic responses in tasks requiring conflict monitoring for stimuli containing incongruent information (e.g., Stroop task; Kerns et al., 2004; Koga et al., 2011). Our behavioral analysis demonstrated that stimulus incongruency prolonged the response time significantly. Still, the effect size of stimulus incongruency (mixed model estimate: +92 ms) was smaller than those of switch (+161 ms), moving leaves (+176 ms), and prior incorrect response (+151 ms; Table 2). Thus, greater iEEG electrode sample sizes at a smaller ROI could have been needed to reveal a significant difference in high-gamma amplitude between incongruent and congruent trials. Further studies involving iEEG oscillations other than high-gamma activity are warranted to determine the network dynamics supporting cognitive flexibility. Prior scalp EEG studies reported that the theta amplitude and coherence across frontal and parietal regions differed between switch and repeat trials (Cooper et al., 2015; Capizzi et al., 2020; McKewen et al., 2021).

Our study also does not rule out the roles of cortico-subcortico-cortical networks in task switching. None of our study patients had iEEG signals recorded from the basal ganglia or thalamus because such spatial sampling was not clinically indicated. Pertinently, a previous lesion study reported that patients with a focal lesion in the basal ganglia frequently failed to perform task switching when needed (Yehene et al., 2008), and an fMRI study of healthy adults reported that requiring a task switch enhanced hemodynamic responses in the basal ganglia (Crone et al., 2006). The mechanistic significance of subcortical structures in task switching may be clarified by future studies measuring event-related high-gamma modulations at depth electrodes implanted in subcortical structures as part of the clinical management of epileptic seizures or movement disorders.

## Supplementary Material

1

2

3

4

5

6

## Figures and Tables

**Table 1:** Patient profile.

Patient number	Age (years)	Gender	Sampled hemisphere	Number of analyzed electrodes	Antiepileptic drugs	Age of epilepsy onset (years)	Seizure onset zone	Pathology
1	11	Male	Left	74	OXC	6	T	Tumor
2	16	Male	Left	100	LEV	9	NA	Tumor
3	20	Male	Right	111	VPA, OXC	6	F	Gliosis
4	11	Male	Left	70	OXC, CLB, LAC	8	T/O	Gliosis
5	9	Male	Left	92	None	9	T	Tumor
6	16	Female	Right	117	CLB, TPM	12	NA	Gliosis
7	14	Female	Right	84	LAC, LTG	5	Insula	Tumor
8	15	Female	Right	113	LTG. ZNS	6	P	Gliosis
9	19	Female	Left	99	OXC	4	F	Gliosis

CLB: Clobazam. LAC: Lacosamide. LEV: Levetiracetam. LTG: Lamotrigine. OXC: Oxcarbazepine. TPM: Topiramate. VPA: Valproate. ZNS: Zonisamide. F: Frontal. O: Occipital. P: Parietal. T: Temporal. NA: not applicable.

**Table 2:** Effects of trial types on response times.

Fixed effect predictors	Mixed model estimate	S.E.	df	t	Pr(> |t|)	95%CI L.L.	U.L.
Switch trial	+161	29	1847.0	5.598	<0.001	+105	+218
Moving trial	+176	23	1848.1	7.806	<0.001	+132	+221
Incongruent trial	+92	22	1848.4	4.117	<0.001	+48	+135
Prior incorrect response	+151	27	1849.9	5.551	<0.001	+98	+205
Log10(trial number)	+13	33	1849.2	0.399	0.690	−52	+79
Game session	−29	8	1850.4	−3.717	<0.001	−44	−14

Dependent variable: Response time (ms). S.E.: Standard error. df: Degree of freedom. Pr: Probability. CI: Confidence interval. L.L.: Lower limit. U.L.: Upper limit. An ancillary mixed model analysis additionally incorporated the interaction of trial types (i.e., [switch × moving], [switch × incongruency], and [moving × incongruency]); it failed to demonstrate a significant interaction between trial types (*p* > 0.05).

**Table 3:** Effects of trial types on response accuracy.

	Proportion of correct response	Chi-square
Switch trial	72.3 (%) (243/336 trials)	5.0
Repeat trial	78.0 (1285/1648)	(*p* = 0.025)
Moving trial	67.3 (673/1000)	119.4
Pointing trial	88.2 (809/917)	*(p <* 0.001)
Incongruent trial	65.9 (615/933)	134.5
Congruent trial	88.1 (867/984)	*(p <* 0.001)
Prior incorrect response	58.2 (262/450)	121.6
Prior correct response	82.9 (1279/1542)	*(p <* 0.001)

## Data Availability

All iEEG data and the MATLAB-based codes used in the analyses are available upon request to the corresponding author.

## Abbreviations:

AUC: area under the curve
CI: confidence interval
DWI: diffusion-weighted imaging
EEG: electroencephalography
ERPs: event-related potentials
FDR: false discovery rate
fMRI: functional magnetic resonance imaging
iEEG: intracranial electroencephalography
IFG: inferior-frontal gyrus
MFG: middle frontal gyrus
MNI: Montreal Neurological Institute
ROC: receiver operating characteristics
ROI: region of interest
STG: superior temporal gyrus
