# Supplementary material for: Temporally and functionally distinct large-scale brain network dynamics supporting task switching
Source: Neuroimage. Author manuscript; Available in PMC 2022 Jul 1. (PMC9173207; doi:10.1016/j.neuroimage.2022.119126)
Supplement: 2 [file NIHMS1811920-supplement-2.docx]

**Ethics statement**

The Institutional Review Ethics Board at Wayne State University has approved the present study. We obtained informed consent from the legal guardians of patients and assent from pediatric patients.
