# Supplementary material for: Temporally and functionally distinct large-scale brain network dynamics supporting task switching
Source: Neuroimage. Author manuscript; Available in PMC 2022 Jul 1. (PMC9173207; doi:10.1016/j.neuroimage.2022.119126)
Supplement: 3 [file NIHMS1811920-supplement-3.docx]

**Supplementary document**

**in**

**Temporally and functionally distinct large-scale brain network dynamics**

**supporting task switching**

Takumi Mitsuhashi; Masaki Sonoda; Ethan Firestone; Kazuki Sakakura;

Jeong-Won Jeong; Aimee F. Luat; Sandeep Sood; Eishi Asano

**This document includes:**

**Figures S1-S6**

**Tables S1-S5**

**Legends for Videos S1-S3**


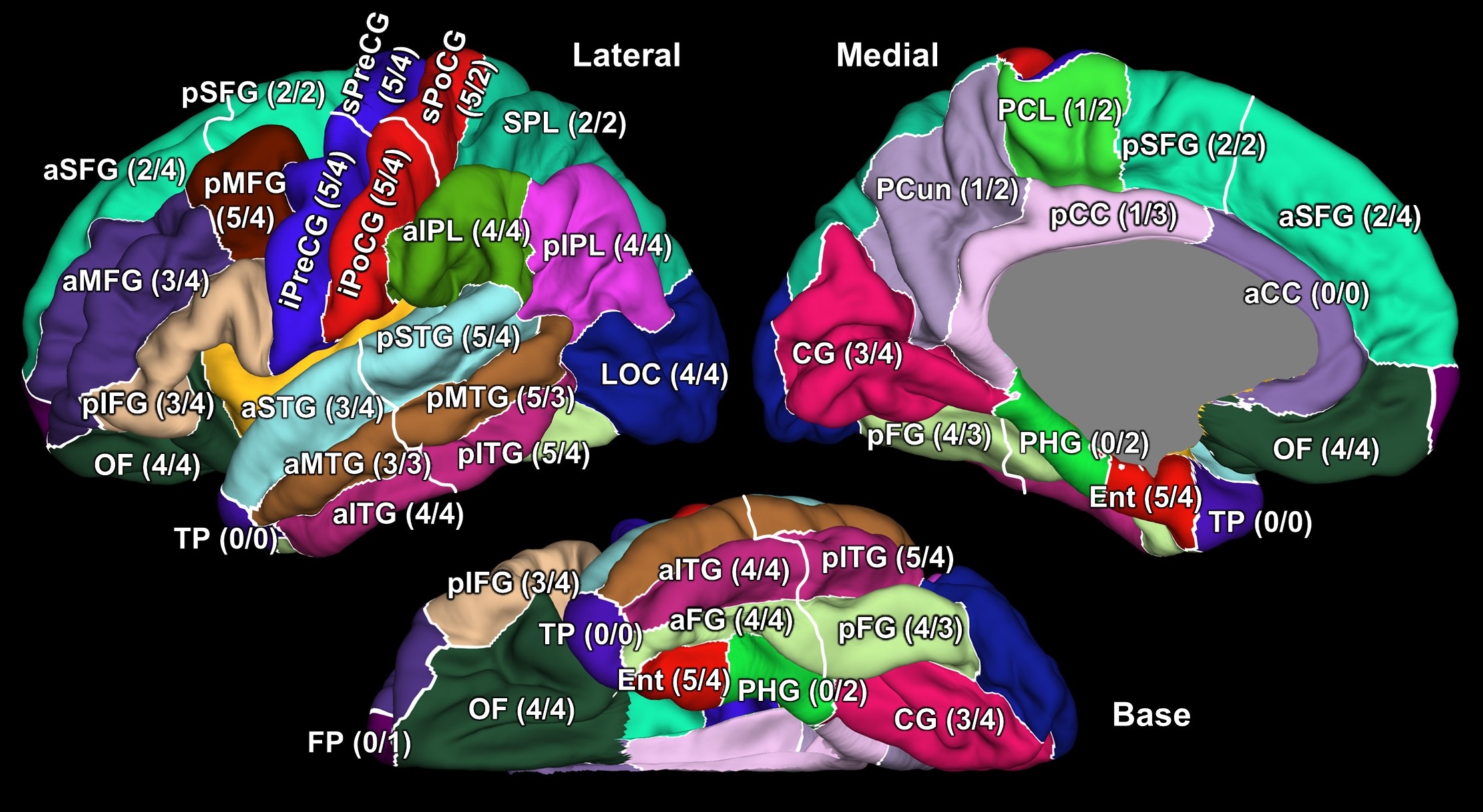


**Fig. S1. Regions of interest (ROI).** aCC = anterior cingulate cortex (Left and Right: 0 and 0 patients); aFG = anterior fusiform gyrus (4 and 4); aIPL = anterior inferior parietal lobule (4 and 4); aITG = anterior inferior temporal gyrus (4 and 4); aMFG = anterior middle frontal gyrus (3 and 4); aMTG = anterior middle temporal gyrus (3 and 3); aSFG = anterior superior frontal gyrus (2 and 4); aSTG = anterior superior temporal gyrus (3 and 4); CG = calcarine gyrus (3 and 4); Ent = entorhinal cortex (5 and 4); FP = frontal pole (0 and 1); iPoCG = inferior postcentral gyrus (5 and 4); iPreCG = inferior precentral gyrus (5 and 4); LOC = lateral occipital cortex (4 and 4); OF = orbitofrontal gyrus (4 and 4); pCC = posterior cingulate cortex (1 and 3); PCL = paracentral lobule (1 and 2); PCun = precuneus (1 and 2); pFG = posterior fusiform gyrus (4 and 3); PHG = parahippocampal gyrus (0 and 2); pIFG = posterior inferior frontal gyrus (3 and 4); pIPL = posterior inferior parietal lobule (4 and 4); pITG = posterior inferior temporal gyrus (5 and 4); pMFG = posterior middle frontal gyrus (5 and 4); pMTG = posterior middle temporal gyrus (5 and 3); pSFG = posterior superior frontal gyrus (2 and 2); pSTG = posterior superior temporal gyrus (5 and 4); SPL = superior parietal lobule (2 and 2); sPoCG = superior postcentral gyrus (5 and 2); sPreCG = superior precentral gyrus (5 and 4); TP = temporal pole (0 and 0). We defined 62 ROIs based on the Desikan FreeSurfer Atlas (Desikan et al., 2006; Nakai et al., 2017); of these, 56 ROIs included electrode sites analyzed in the present study. The CG was defined as the summation of lingual, cuneus, and peri-calcarine gyri. In each parenthesis, we have provided the number of patients who had iEEG electrodes at given left and right-hemispheric ROIs.

**
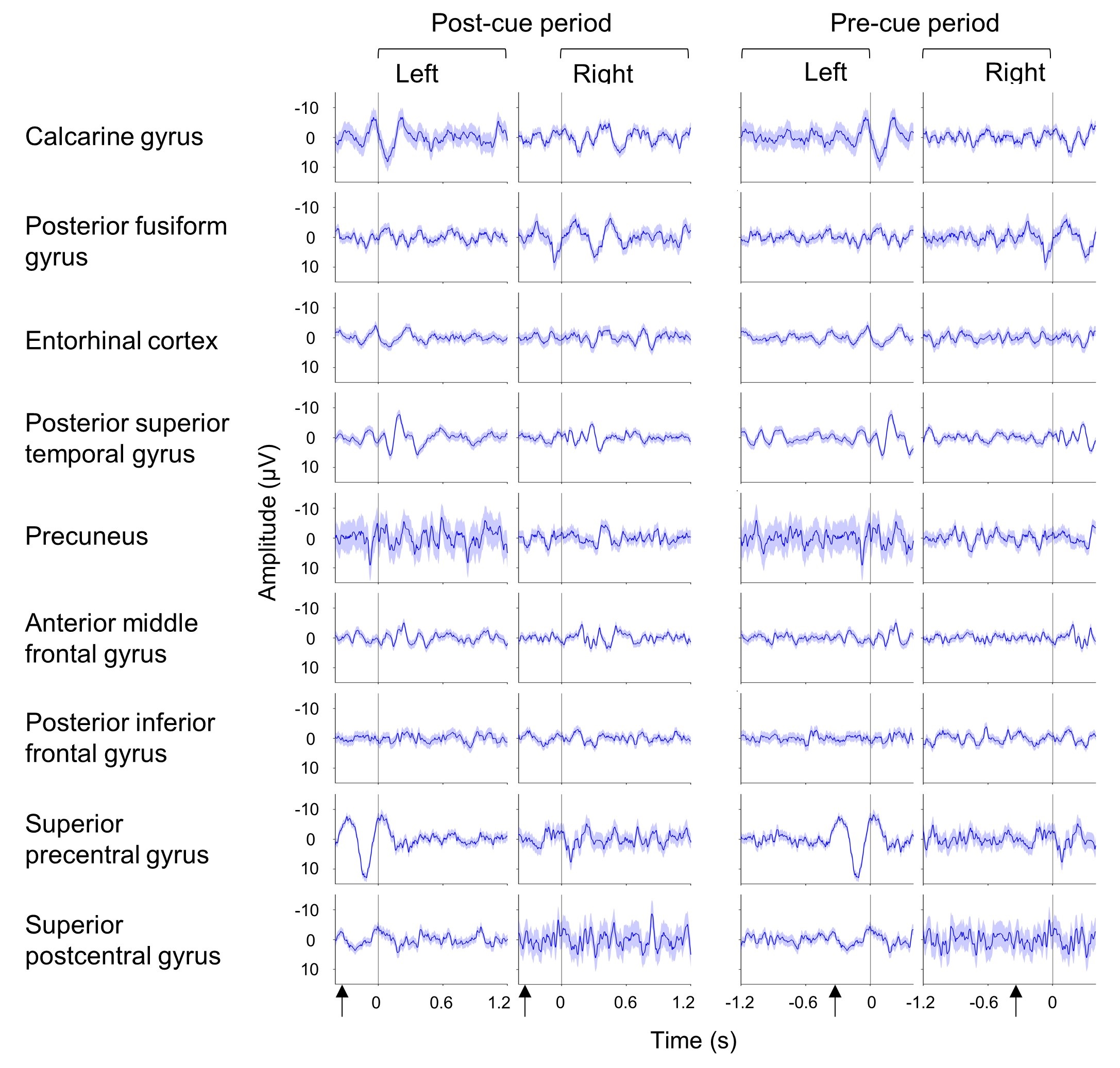
**

**Fig. S2. Broadband evoked responses during the Lumosity gameplay.** We computed broadband evoked responses (also known as event-related potentials) by averaging iEEG signals with a bandpass of 3-110 Hz, at given electrode sites, time-locked to the task cue onset (i.e., feedback sound onset/response detection). The pre-cue period is referred to as the pre-response detection period. Solid line: mean broadband evoked response across all available electrode sites within a given region of interest. Blue shade: 95% confidence interval. Arrow: Estimated screen tapping onset (333 ms before the task cue onset).


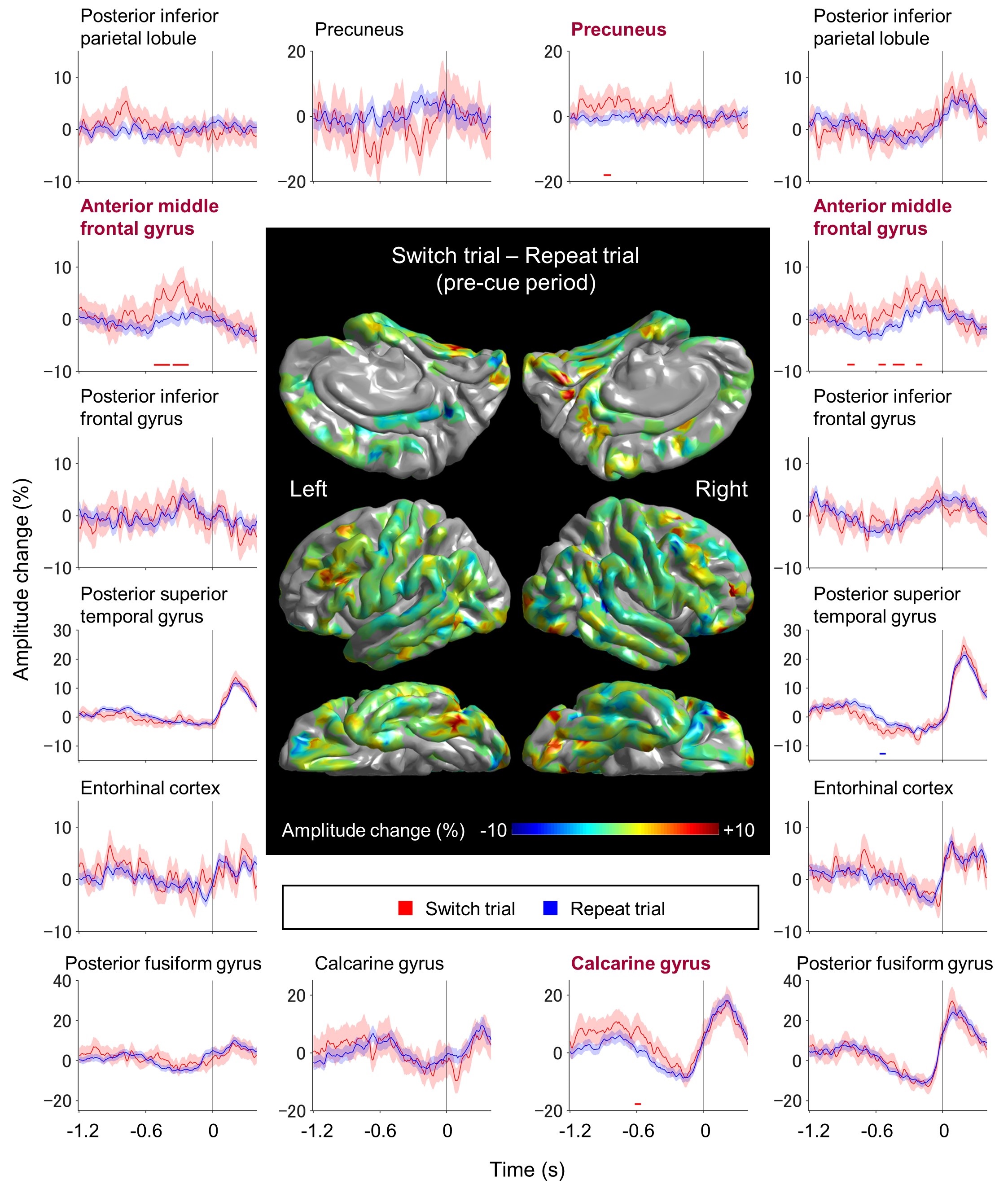


**Fig. S3. The dynamics of high-gamma modulations in switch and repeat trials (pre-cue period).** The brain surface map shows the subtraction of high-gamma amplitudes during the 1,200-ms pre-cue period of repeat trials from those of switch trials. The pre-cue period can be referred to as the pre-response detection period. Plots present the dynamics of high-gamma modulations at given regions of interest. Red lines: switch trials. Blue lines: repeat trials. Shading: 95% confidence interval. High-gamma values presented herein are amplitude changes compared to the average during the 1,200-ms pre-cue period. The zero-time point: task cue onset. With the inherent processing latency of the gameplay platform on our iPad, the screen tapping onset was estimated to be 333 ms before the zero-time point. Horizontal bar: time windows showing a significant difference in high-gamma amplitudes between switch and repeat trials based on the permutation test.

**
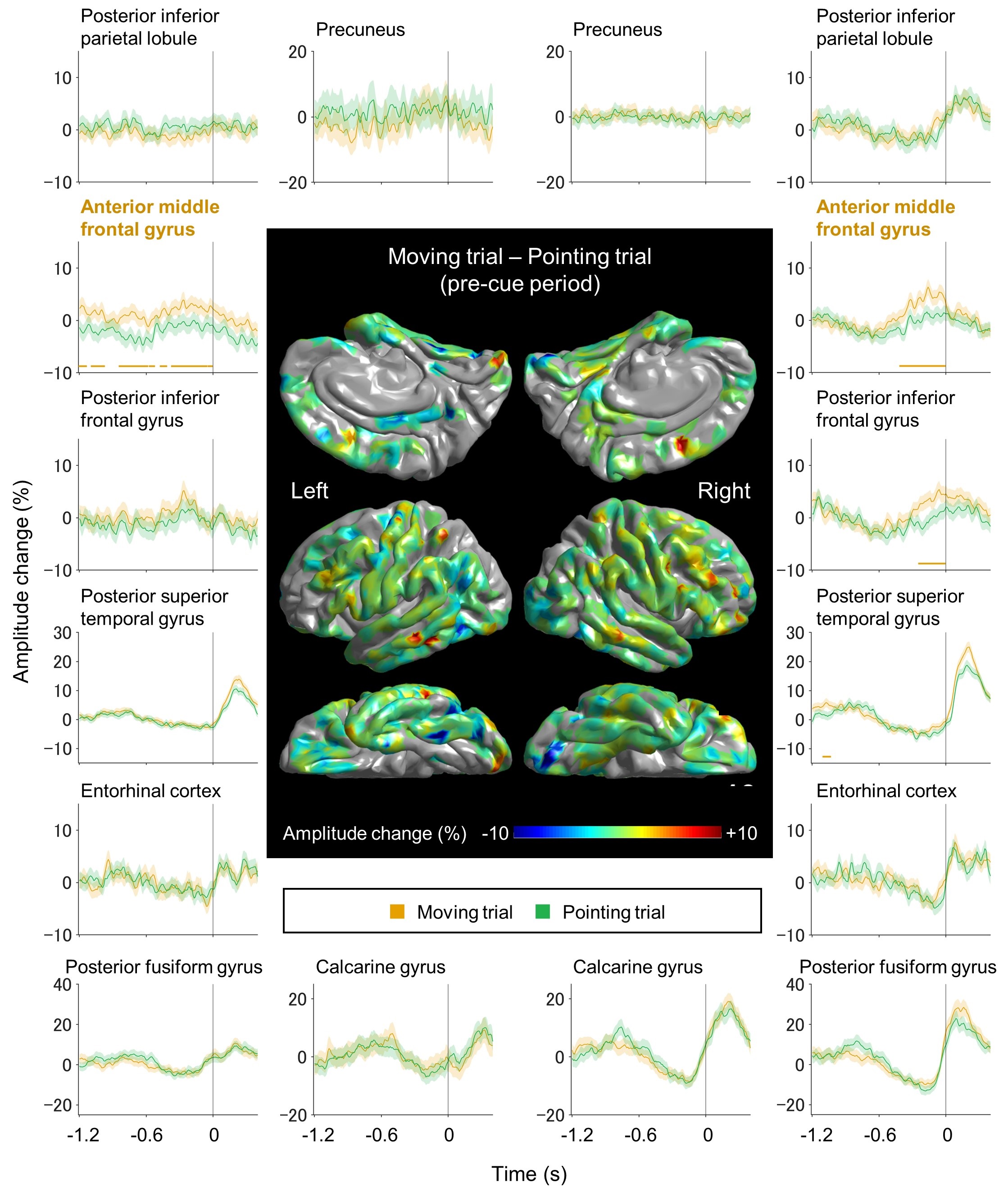
**

**Fig. S4. The dynamics of high-gamma modulations in moving and pointing trials (pre-cue period).** The brain surface map shows the subtraction of high-gamma amplitudes during the 1,200-ms pre-cue period of pointing trials from those of moving trials. Plots present the dynamics of high-gamma modulations at given regions of interest. Orange lines: moving trials. Green lines: pointing trials. Shading: 95% confidence interval. High-gamma values presented herein are amplitude changes compared to the average during the 1,200-ms pre-cue period. The zero-time point: task cue onset. With the inherent processing latency of the gameplay platform on our iPad, the screen tapping onset was estimated to be 333 ms before the zero-time point. Horizontal bar: time windows showing a significant difference in high-gamma amplitudes between moving and pointing trials based on the permutation test.

**
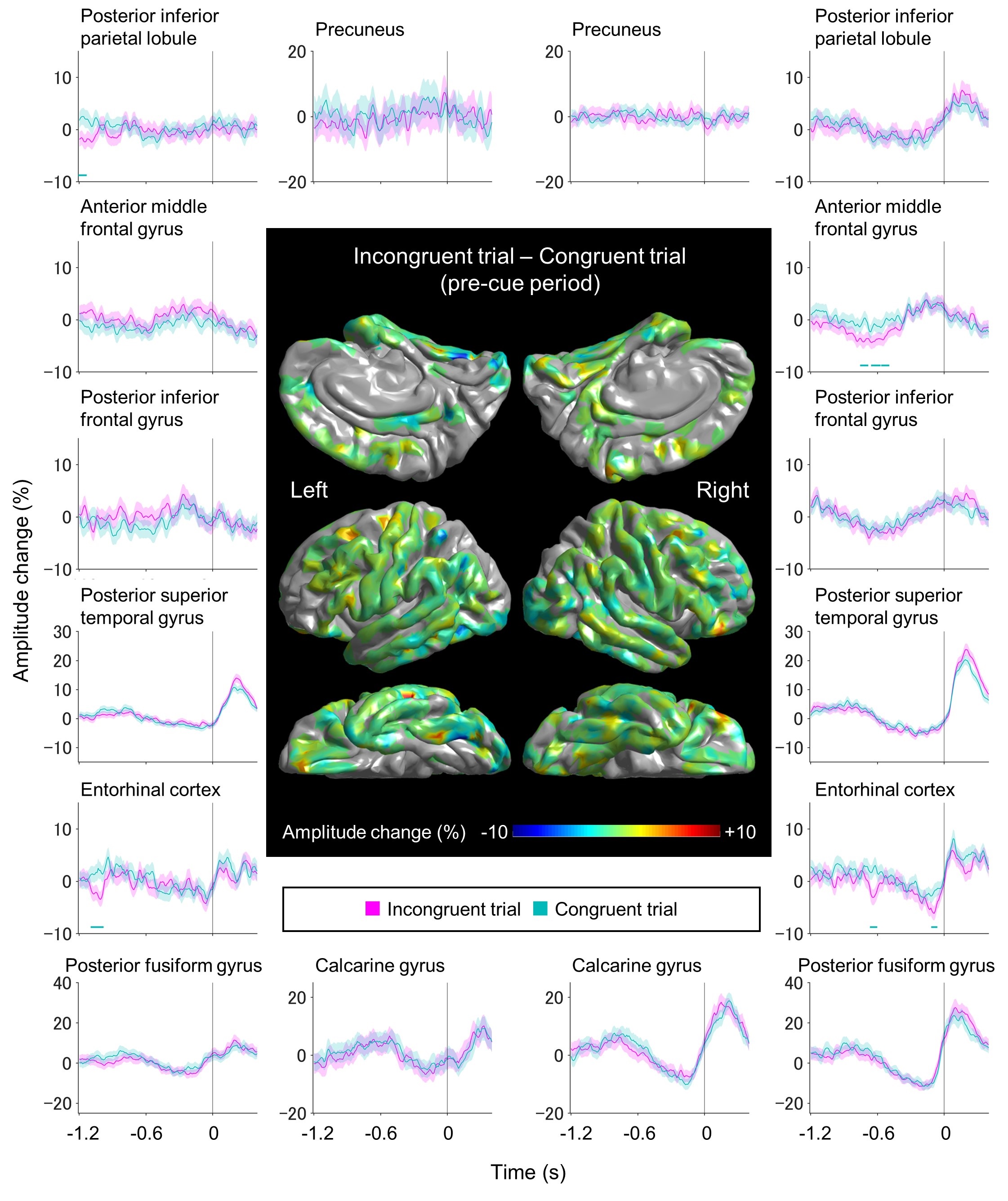
**

**Fig. S5. The dynamics of high-gamma modulations in incongruent and congruent trials (pre-cue period).** The brain surface map shows the subtraction of high-gamma amplitudes during the 1,200-ms pre-cue period of congruent trials from those of incongruent trials. Plots present the dynamics of high-gamma modulations at given regions of interest. Magenta lines: incongruent trials. Cyan lines: congruent trials. Shading: 95% confidence interval. High-gamma values presented herein are amplitude changes compared to the average during the 1,200-ms pre-cue period. The zero-time point: task cue onset. With the inherent processing latency of the gameplay platform on our iPad, the screen tapping onset was estimated to be 333 ms before the zero-time point. Horizontal bar: time windows showing a significant difference in high-gamma amplitudes between incongruent and congruent trials based on the permutation test.

**
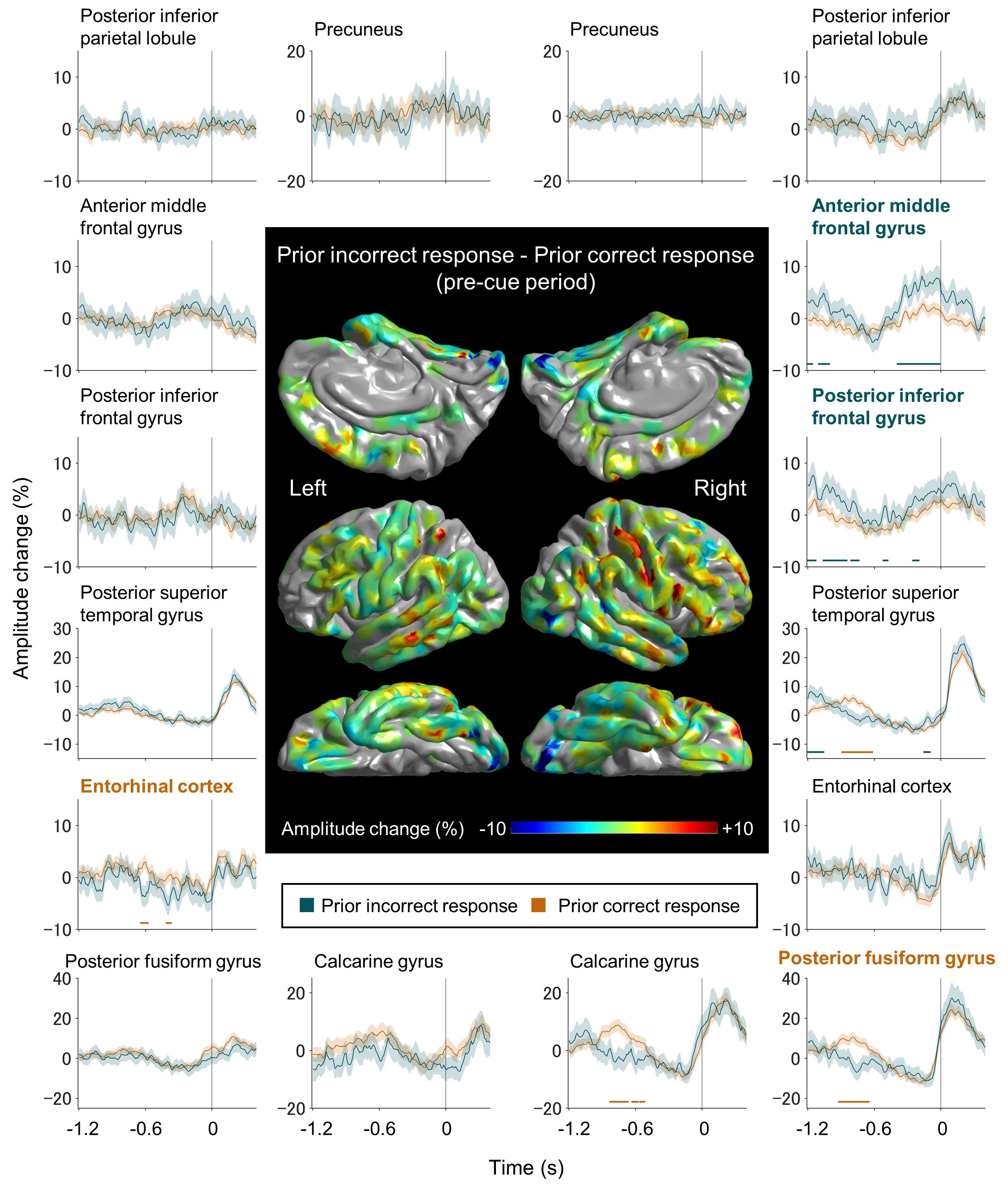
**

**Fig. S6. The dynamics of high-gamma modulations in trials preceded by an incorrect and correct response (pre-cue period).** The brain surface map shows the subtraction of high-gamma amplitudes during the 1,200-ms pre-cue period of trials preceded by a correct response from those of trials preceded by an incorrect response. Dark green lines: trials preceded by an incorrect response. Brown lines: trials preceded by a correct response. High-gamma values presented herein are amplitude changes compared to the average during the 1,200-ms pre-cue period. The zero-time point: task cue onset. The screen tapping onset was estimated to be 333 ms before the zero-time point. Horizontal bar: time windows showing a significant difference in high-gamma amplitudes between trials preceded by an incorrect and correct response based on the permutation test.

**Table S1. Effects of trial types on post-cue high-gamma amplitudes in the right calcarine gyrus.**

| Fixed effect predictors | Mixed model estimate | S.E. | df | t | Pr(>\|t\|) | 95%CI | |
| --- | --- | --- | --- | --- | --- | --- | --- |
|  |  |  |  |  |  | L.L. | U.L. |
| Switch trial | +4.03 | 0.94 | 3416 | +4.271 | **< 0.001** | +2.18 | +5.87 |
| Moving trial | -0.29 | 0.74 | 3416 | -0.390 | 0.8868 | -1.73 | +1.16 |
| Incongruent trial | +1.12 | 0.73 | 3416 | +1.535 | 0.3506 | -0.31 | +2.55 |
| Prior incorrect response | -0.83 | 0.92 | 3416 | -0.892 | 0.5346 | -2.64 | +0.99 |
| Log10(trial number) | -5.63 | 0.96 | 3416 | -5.845 | **< 0.001** | -7.51 | -3.74 |
| Game session | -0.33 | 0.27 | 3416 | -2.481 | **0.047** | -0.59 | -0.06 |

Dependent variable: high-gamma amplitudes (%). S.E.: Standard error. df: Degree of freedom. Pr: Probability. CI: Confidence interval. L.L.: Lower limit. U.L.: Upper limit.

**Table S2. Effects of trial types on post-cue high-gamma amplitudes in the right anterior middle frontal gyrus.**

| Fixed effect predictors | Mixed model estimate | S.E. | df | t | Pr(>\|t\|) | 95%CI | |
| --- | --- | --- | --- | --- | --- | --- | --- |
|  |  |  |  |  |  | L.L. | U.L. |
| Switch trial | +1.58 | 0.51 | 5654 | +3.117 | **0.013** | +0.59 | +2.58 |
| Moving trial | +0.95 | 0.39 | 5654 | +2.402 | 0.065 | +0.17 | +1.72 |
| Incongruent trial | -0.20 | 0.39 | 5654 | -0.508 | 0.835 | -0.97 | +0.57 |
| Prior incorrect response | +3.83 | 0.49 | 5654 | +7.864 | **< 0.001** | +2.88 | +4.79 |
| Log10(trial number) | -1.91 | 0.52 | 5654 | -3.664 | **< 0.001** | -2.93 | -0.89 |
| Game session | -0.71 | 0.14 | 5654 | -4.988 | **< 0.001** | -0.99 | -0.43 |

Dependent variable: high-gamma amplitudes (%). S.E.: Standard error. df: Degree of freedom. Pr: Probability. CI: Confidence interval. L.L.: Lower limit. U.L.: Upper limit.

**Table S3. Effects of trial types on post-cue high-gamma amplitudes in the right entorhinal cortex.**

| Fixed effect predictors | Mixed model estimate | S.E. | df | t | Pr(>\|t\|) | 95%CI | |
| --- | --- | --- | --- | --- | --- | --- | --- |
|  |  |  |  |  |  | L.L. | U.L. |
| Switch trial | +1.33 | 0.39 | 4404 | +3.445 | **0.008** | +0.57 | +2.09 |
| Moving trial | -0.31 | 0.30 | 4404 | -1.013 | 0.600 | -0.90 | +0.29 |
| Incongruent trial | +0.73 | 0.30 | 4404 | +2.418 | 0.175 | +0.14 | +1.32 |
| Prior incorrect response | +0.05 | 0.38 | 4404 | +0.131 | 0.912 | -0.70 | +0.80 |
| Log10(trial number) | -0.29 | 0.40 | 4404 | -0.720 | 0.562 | -1.06 | +0.49 |
| Game session | -0.73 | 0.11 | 4404 | -6.704 | **< 0.001** | -0.94 | -0.52 |

Dependent variable: high-gamma amplitudes (%). S.E.: Standard error. df: Degree of freedom. Pr: Probability. CI: Confidence interval. L.L.: Lower limit. U.L.: Upper limit.

**Table S4. Effects of trial types on post-cue high-gamma amplitudes in the left anterior middle frontal gyrus.**

| Fixed effect predictors | Mixed model estimate | S.E. | df | t | Pr(>\|t\|) | 95%CI | |
| --- | --- | --- | --- | --- | --- | --- | --- |
|  |  |  |  |  |  | L.L. | U.L. |
| Switch trial | +3.02 | 0.59 | 3849 | +5.134 | **< 0.001** | +1.86 | +4.17 |
| Moving trial | +1.21 | 0.46 | 3849 | +2.646 | **0.038** | +0.31 | +2.11 |
| Incongruent trial | +0.62 | 0.45 | 3849 | +1.371 | 0.378 | -0.27 | +1.50 |
| Prior incorrect response | +0.51 | 0.55 | 3849 | +0.917 | 0.530 | -0.58 | +1.59 |
| Log10(trial number) | -0.47 | 0.59 | 3849 | -0.804 | 0.525 | -1.63 | +0.68 |
| Game session | -0.58 | 0.15 | 3849 | -3.783 | **< 0.001** | -0.88 | -0.28 |

Dependent variable: high-gamma amplitudes (%). S.E.: Standard error. df: Degree of freedom. Pr: Probability. CI: Confidence interval. L.L.: Lower limit. U.L.: Upper limit.

**Table S5. Effects of trial types on post-cue high-gamma amplitudes in the right precuneus.**

| Fixed effect predictors | Mixed model estimate | S.E. | df | t | Pr(>\|t\|) | 95%CI | |
| --- | --- | --- | --- | --- | --- | --- | --- |
|  |  |  |  |  |  | L.L. | U.L. |
| Switch trial | +3.75 | 0.72 | 1175 | +5.247 | **< 0.001** | +2.35 | +5.16 |
| Moving trial | +0.02 | 0.55 | 1175 | +0.044 | 0.965 | -1.06 | +1.11 |
| Incongruent trial | -0.83 | 0.56 | 1175 | -1.485 | 0.351 | -1.92 | +0.27 |
| Prior incorrect response | -0.39 | 0.67 | 1175 | -0.588 | 0.701 | -1.71 | +0.92 |
| Log10(trial number) | +2.17 | 0.73 | 1175 | +2.958 | **0.003** | +0.73 | +3.60 |
| Game session | -0.02 | 0.20 | 1175 | -0.087 | 0.948 | -0.40 | +0.37 |

Dependent variable: high-gamma amplitudes (%). S.E.: Standard error. df: Degree of freedom. Pr: Probability. CI: Confidence interval. L.L.: Lower limit. U.L.: Upper limit.

**Video Legends**

**Video S1. The task-switching paradigm.**

An author (T.M.) demonstrates how to play the game: *Ebb and Flow*, a cognitive flexibility training game on the Lumosity platform. The video presents examples of task cues. When the leaf color was green, each player must swipe the screen in the direction the leaf was pointing. When the leaf color was orange, each player must swipe in the direction the leaf was moving. When the leaves’ pointing did not match their moving direction, given trials were considered incongruent. The task rule (i.e., leave color) was switched unpredictably. Note that the total number of completed trials during five-minute gameplay was 221.4 per patient on average (range: 171-285).

**Video S2. Spatiotemporal dynamics of high-gamma modulations averaged across all trials.**

The animation video demonstrates the spatiotemporal dynamics of high-gamma modulations during the Lumosity gameplay. We computed the percent change of high-gamma amplitude compared to the mean amplitude during the *non-gameplay* reference period. The zero-time point: onset of task cue (i.e., response detection = feedback sound onset). With the inherent processing latency of the gameplay platform on our iPad, the screen tapping onset was estimated to be 333 ms, on average, before the zero-time point (95% confidence interval: 325 ms to 341 ms).

**Video S3. Spatiotemporal dynamics of gameplay-related high-gamma modulations.**

The animation video demonstrates the spatiotemporal dynamics of high-gamma modulations during the Lumosity gameplay. We computed the percent change of high-gamma amplitude compared to the mean during each 1,200-ms *gameplay* period. The zero-time point: onset of task cue (i.e., response detection = feedback sound onset). With the inherent processing latency of the gameplay platform on our iPad, the screen tapping onset was estimated to be 333 ms, on average, before the zero-time point (95% confidence interval: 325 ms to 341 ms).

**Supplementary Reference**

[S1] Desikan, R.S., Segonne, F., Fischl, B., Quinn, B.T., Dickerson, B.C., Blacker, D., Buckner, R.L., Dale, A.M., Maguire, R.P., Hyman, B.T., Albert, M.S., Killiany, R.J., 2006. An automated labeling system for subdividing the human cerebral cortex on MRI scans into gyral based regions of interest. Neuroimage 31(3),968-980. https://doi.org/10.1016/j.neuroimage.2006.01.021.

[S2] Nakai, Y., Jeong, J.W., Brown, E.C., Rothermel, R., Kojima, K., Kambara, T., Shah, A., Mittal, S., Sood, S., Asano, E., 2017. Three- and four-dimensional mapping of speech and language in patients with epilepsy. Brain 140(5),1351-1370. https://doi.org/10.1093/brain/awx051.
